# The role of mitochondria in aging, cell death, and tumor immunity

**DOI:** 10.3389/fimmu.2024.1520072

**Published:** 2024-12-23

**Authors:** Qiang Wang, Yixiao Yuan, Jing Liu, Chunhong Li, Xiulin Jiang

**Affiliations:** ^1^ Gastrointestinal Surgical Unit, Suining Central Hospital, Suining, Sichuan, China; ^2^ Department of Medicine, University of Florida (UF) Health Cancer Center, University of Florida, Gainesville, FL, United States; ^3^ Department of Oncology, Suining Central Hospital, Suining, Sichuan, China

**Keywords:** mitochondria, metabolism, aging, cell death, cancer, TME

## Abstract

Mitochondria are essential double-membrane organelles with intricate structures and diverse functions within cells. Under normal physiological conditions, mitochondria regulate cellular metabolism and maintain energy homeostasis via the electron transport chain, mediate stem cell fate, and modulate reactive oxygen species production, playing a pivotal role in energy supply and lifespan extension. However, mitochondrial dysfunction can lead to various pathological changes, including cellular aging, necrosis, dysregulated tumor immunity, and the initiation and progression of cancer. Moreover, abnormal mitochondrial metabolism is closely associated with numerous diseases, such as neurodegenerative disorders, metabolic syndromes, and cancers. In recent years, targeting mitochondria has emerged as a promising anticancer strategy, aiming to modulate mitochondrial functions and metabolism for therapeutic benefits. Nonetheless, such approaches face limitations, including low delivery efficiency and insufficient specificity. This review systematically explores mitochondrial structure and function, their physiological and pathological roles, and the potential and challenges of mitochondria-targeted strategies in cancer therapy, providing insights for future research directions.

## Introduction

As vital organelles in eukaryotic cells, mitochondria are involved in regulating cellular metabolism, health and longevity, cell death, tumor microenvironment, autophagy, and cell fate (1). Mitochondria evolved from prokaryotic α-proteobacteria and are present in most eukaryotic cells, consisting of five distinct structures: the outer mitochondrial membrane, inner mitochondrial membrane, intermembrane space, cristae, and matrix (2). Mitochondria are the only organelles in animal cells, besides the nucleus, that contain DNA. Mitochondrial DNA (mtDNA) is a circular DNA that encodes 13 proteins essential for oxidative phosphorylation complex formation, 22 transfer RNAs, and 2 ribosomal RNAs necessary for mitochondrial RNA translation (3, 4). As the center of cellular energy metabolism and biosynthesis, mitochondria produce ATP through oxidative phosphorylation to maintain cellular energy homeostasis (5), and their metabolic intermediates are crucial participants in biosynthetic pathways (6). Additionally, mitochondria regulate various cellular biological processes, including energy metabolism, cell fate determination, and immune responses, by releasing mtDNA, mtROS, and metabolic products. Increasing evidence indicates that mitochondrial dysfunction is associated with various diseases, including autoimmune diseases (7), and dysfunctional mitochondria drive cellular malignant transformation (8). Beyond their role in energy metabolism, mitochondria also regulate cell fate and function by modulating the release of different signaling molecules. CoAs, as important metabolic intermediates, mainly participate in chromatin remodeling as substrates for histone lysine acylation, and TCA cycle metabolites can act as substrates for histone-modifying enzymes to regulate gene expression through histone modifications (9). Calcium ions, as second messengers within cells, are also regulated by mitochondria (10). Mitochondria control calcium ion flux from the plasma membrane and endoplasmic reticulum, thus regulating the spatial and temporal distribution of calcium ions within cells (11). As a central hub for cell death and inflammation regulation, mitochondria control the release of cytochrome c (cyt c), initiating caspase-dependent cell death and inflammation-related cascade reactions (12). Reactive oxygen species (ROS) within cells can damage proteins, lipids, and DNA. The mitochondrial electron transport chain, as a major site of ROS production, is involved in cancer progression, cardiovascular diseases, and neurodegenerative diseases (13). Despite being by-products of metabolic processes, excessive ROS levels can harm cells. Nevertheless, intracellular ROS also plays a role in stem cell fate transitions. Embryonic stem cells and induced pluripotent stem cells maintain genomic integrity by sustaining low levels of endogenous ROS, while moderate increases in ROS are necessary for stem cells to differentiate into various lineages (14).

Tumor cells adapt to increasing energy and biosynthetic demands by reprogramming related metabolic pathways. In the tumor microenvironment, nutrient depletion and excessive production of metabolic by-products driven by tumor development regulate the metabolic reprogramming of tumor-infiltrating immune cells and activate related signals to control the polarization of different types of immune cells, inducing metabolic disorder-mediated anti-tumor immune response failure and helping establish an immune-suppressive tumor microenvironment. Mitochondria, as highly dynamic organelles with various biological functions within cells (15), play a key regulatory role in metabolism and immune cell activation. Studies have shown that mitochondrial dysfunction in various cells within the tumor microenvironment, including tumor cells and immune cells, is a crucial factor in cancer initiation, progression, and metastasis (16). This paper summarizes mitochondrial structural components, genomic features, and the role of mitochondria in regulating cellular metabolism, stem cell fate regulation, and longevity. Additionally, we explore the relationship between mitochondria in regulating cell death, aging, and tumor immune microenvironment. We discuss the role of mitochondria in immune cell activation, the regulatory role of key mitochondrial components mtDNA and mtROS in tumor initiation, progression, immune-suppressive tumor microenvironment formation, and tumor immune evasion. We also examine the dual role of mitophagy in tumor progression, aiming to deepen the understanding of the core role of mitochondria in tumor development and to provide a basis for developing mitochondrial-targeted anti-tumor therapies.

To guide readers through this manuscript, we provide the following structural roadmap: first, we outline the structural composition of mitochondria and their pivotal roles in normal physiological functions, including metabolic regulation, energy production via the electron transport chain, stem cell fate determination, reactive oxygen species (ROS) generation, and their impact on longevity. Next, we focus on the pathological consequences of mitochondrial dysfunction, such as cellular aging, necrosis, dysregulated tumor immunity, and the mechanisms underlying cancer progression. Subsequently, we analyze the strong association between abnormal mitochondrial metabolism and various diseases, including neurodegenerative disorders, metabolic syndromes, and cancer. Following this, we discuss the potential of mitochondria-targeted strategies in cancer therapy, highlighting their advantages as well as current challenges, such as delivery efficiency and specificity. Finally, we summarize the progress in this field and propose future research directions and potential solutions to advance the understanding of mitochondrial functions, offering new perspectives for further investigation.

## Composition and physiological functions of mitochondria

Mitochondria provide the majority of the energy required for life activities and are thus considered the “powerhouses” of cells (17). Mitochondria consist of two layers of unit membranes composed mainly of lipids and proteins. The physicochemical properties of these special lipids and proteins (such as charge distribution) promote membrane curvature and fluidity, compartmentalizing the mitochondria into multiple aqueous cavities, leading to the concept of “mitochondrial compartmentalized structure.” The outer membrane is in direct contact with the cytosol, while the inner membrane can be further divided into the inner boundary membrane, adjacent to the outer membrane, and the cristae membrane, projecting into the mitochondrial matrix (18). The space between the outer membrane and the inner boundary membrane is known as the intermembrane space; the spaces between the cristae are referred to as the Intracoastal space; and the innermost cavity of the mitochondria is the matrix. The cristae themselves can be subdivided into functional regions, including crista junctions connecting the cristae membrane to the inner boundary membrane and the crista tips at the distal ends. Temporary contact points between the inner and outer membranes, called translocation contact sites, house protein complexes such as the mitochondrial contact site and cristae organizing system (MICOS) and translocases of the inner and outer membranes (TIM and TOM complexes) (19). The dynamic changes in the mitochondrial inner and outer membranes, along with the different distributions of macromolecules and biochemical processes occurring in various mitochondrial compartments, are linked to mitochondrial dysfunction, which is associated with aging and cancer (20).

**Figure 1:**
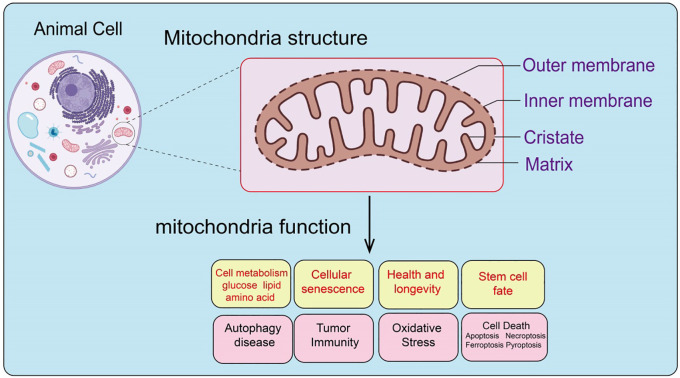
Structural composition and function of mitochondria.

Functionally, mitochondria serve as the core of cellular energy metabolism, generating ATP through oxidative phosphorylation to provide energy for cellular activities. In addition, mitochondria play crucial roles in regulating cell growth, differentiation, aging, and apoptosis (21). Mitochondria are also vital in sustaining cellular metabolic homeostasis, regulating immune responses, and adapting to various stress conditions (22). Therefore, mitochondria are not only the powerhouses of the cell but also critical hubs in determining cell fate (**Figure 1**).

Unlike other organelles, the number of mitochondria within a cell varies, and the total mitochondrial content in a cell is referred to as the “mitochondrial pool.” Mitochondria are maternally inherited and possess their own genetic system and protein translation system (23). Mitochondrial DNA (mtDNA) is a circular DNA molecule composed of 16,568 base pairs, divided into heavy and light strands (24). It primarily encodes 13 protein subunits of the electron transport chain, two rRNAs (16S and 12S), and 22 tRNAs. Unlike diploid nuclear DNA, mtDNA is polyploid, with tens to thousands of copies per cell, and healthy cells exhibit relatively low levels of mtDNA mutations (20). mtDNA attaches to the inner mitochondrial membrane, close to the electron transport chain (ETC), and in contact with reactive oxygen species (ROS). High levels of ROS are associated with increased mtDNA mutation load and tumor progression, also impairing the function of DNA polymerase γ, leading to replication errors in mtDNA (25). Due to the lack of protective histones and limited error correction mechanisms, the mtDNA mutation rate is 100 times higher than that of nuclear DNA. mtDNA mutations have been observed to expand and increase over time (26). Sequence changes in mtDNA are common in various tumors, significantly influencing tumorigenesis and progression by affecting mitochondrial ROS production and redox status (27).

## Mitochondria and cellular energy metabolism

**Figure 2:**
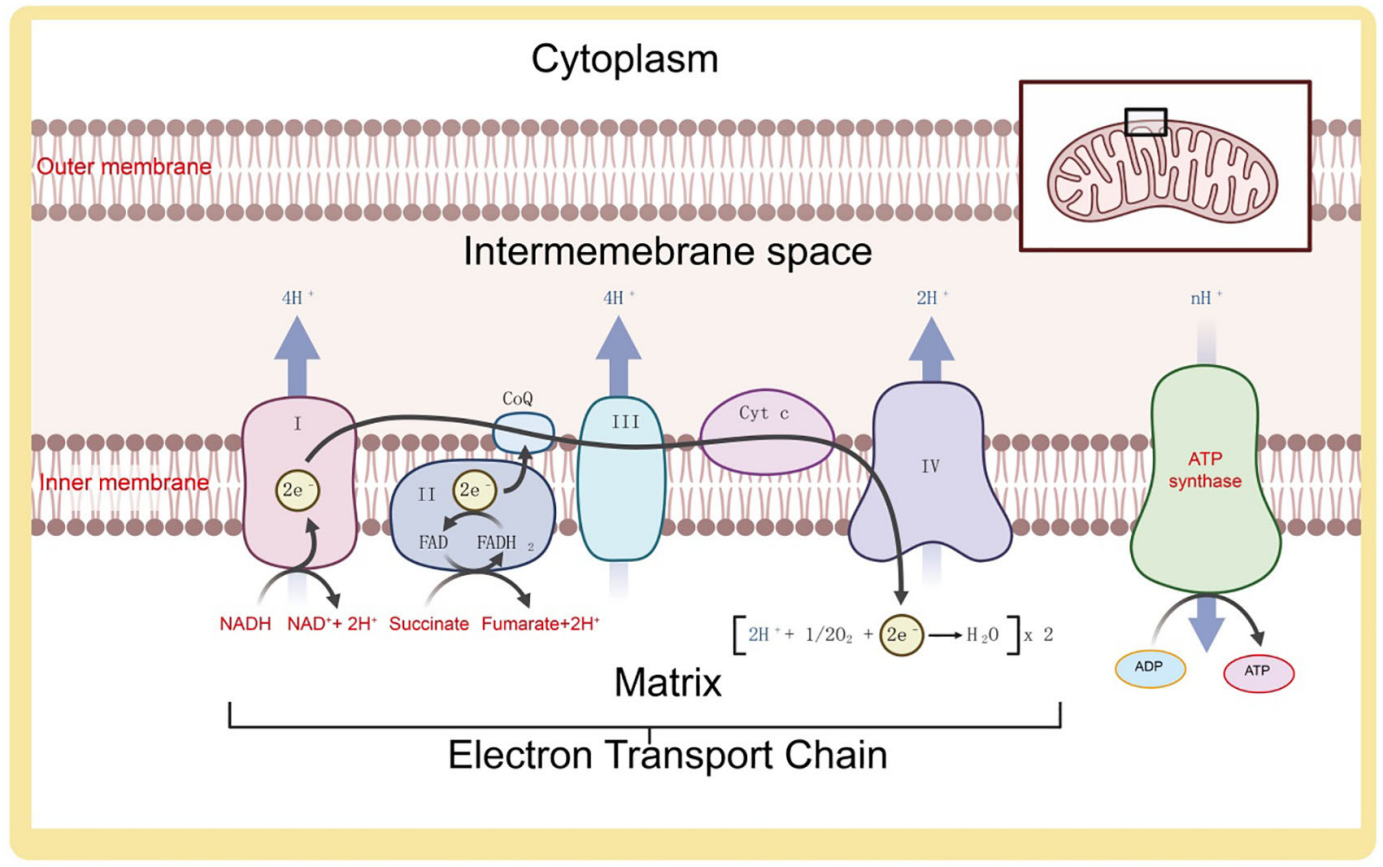
Composition and function of the electron transport chain on the mitochondrial inner membrane.

Mitochondria are key organelles in cellular energy metabolism, primarily involved in regulating energy through glycolysis, the tricarboxylic acid (TCA) cycle, and oxidative phosphorylation (28). In the cytosol, phosphorylated glucose substrates undergo glycolysis to produce pyruvate, which is further oxidized to acetyl-CoA and enters the TCA cycle. Glycolysis and the TCA cycle generate reduced electron carriers, NADH, which, under the action of dehydrogenases, release high-energy electrons into the mitochondrial inner membrane’s ETC (14). Through successive redox reactions, the ETC undergoes conformational changes, consumes oxygen, and transfers protons, creating a proton gradient between the mitochondrial intermembrane space and the matrix, thereby driving ATP synthesis (29). Mitochondrial metabolic reprogramming is a key process for cells to adapt to environmental changes, cope with stress, and support rapid proliferation. Studies have shown that cancer cells maintain high energy demand and support tumor growth by altering the balance between oxidative phosphorylation (OXPHOS) and glycolysis. Particularly in the tumor microenvironment, hypoxic conditions drive cancer cells to favor glycolysis through the “Warburg effect,” even when oxygen supply is sufficient. Additionally, mitochondrial metabolic pathways, such as fatty acid oxidation and amino acid metabolism, also play crucial roles in various types of cancer. Under hypoxic conditions, cells primarily generate energy through anaerobic respiration, where glycolysis-produced pyruvate is not funneled into the mitochondrial TCA cycle but is instead enzymatically converted into lactate as a less efficient energy source (30) (**Figure 2**).

## Formation of mitochondrial reactive oxygen species and antioxidant defense

**Figure 3:**
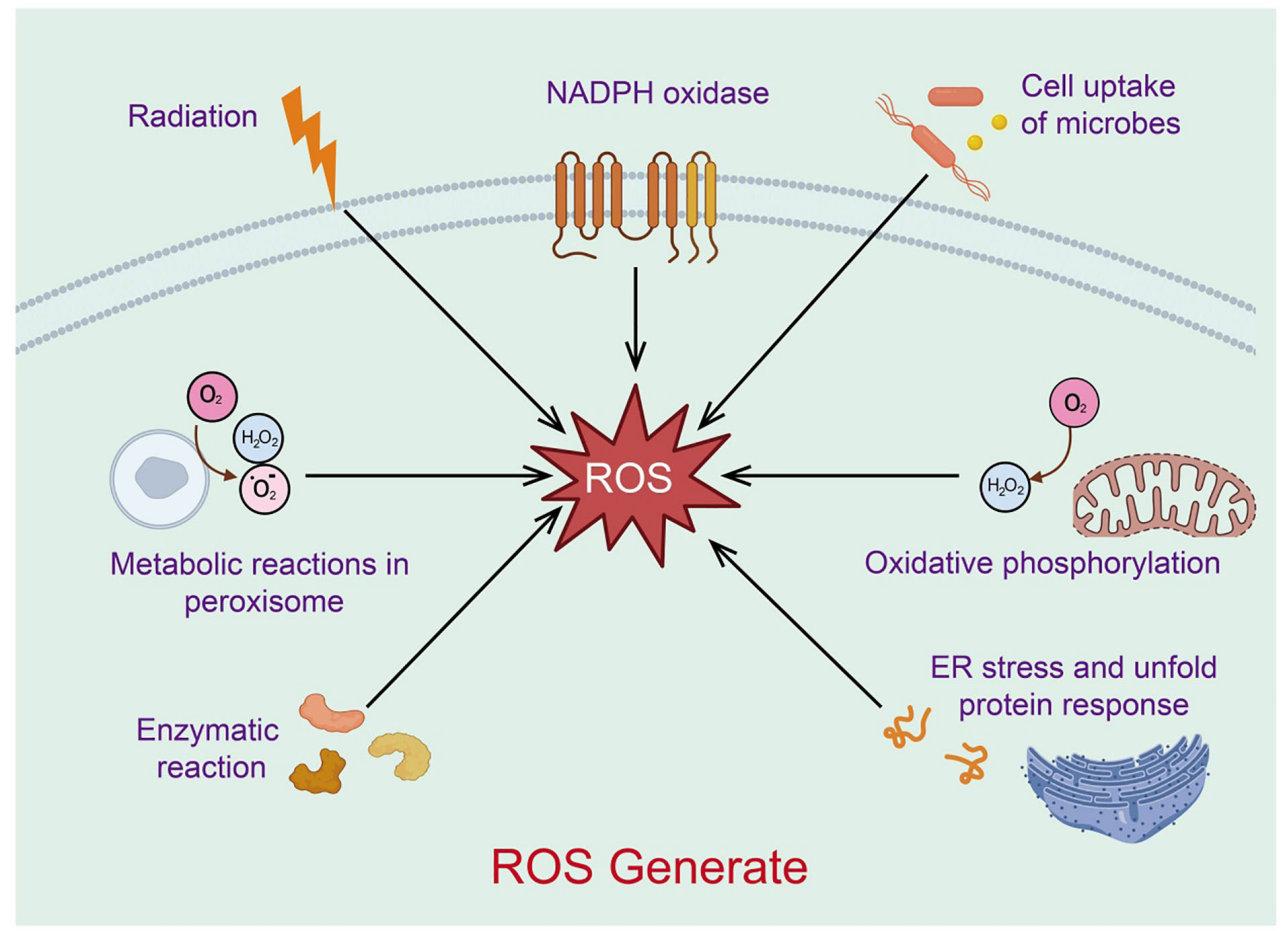
Sources of mitochondria-associated reactive oxygen species.

Reactive oxygen species (ROS) are a class of highly reactive oxygen-containing radicals produced by aerobic cells during metabolism, including superoxide anions, hydrogen peroxide (H_2_O_2_), and hydroxyl radicals. Mitochondria are the primary source of ROS (31). As the cellular organelles responsible for energy production, mitochondria oxidize glucose, fatty acids, and amino acid metabolites to generate ATP through oxidative phosphorylation and the Krebs cycle (32). In this process, most electrons provided to the respiratory chain react with protons and oxygen, forming H_2_O at the level of cytochrome c oxidase (or Complex IV). These superoxide anion radicals quickly convert to H_2_O_2_, which can further react with divalent iron to produce hydroxyl radicals. The superoxide anion radicals generated in this process are an important source of ROS (33). Low levels of mitochondrial ROS appear to be crucial signaling factors for maintaining cellular homeostasis and inducing stress responses. Excessive mitochondrial ROS, resulting from mitochondrial dysfunction, can further damage mitochondrial DNA, proteins, and lipids, promoting more ROS production and creating a vicious cycle (34) (**Figure 3**). Thus, mitochondria act as both regulators and mediators of ROS, balancing cellular survival and apoptosis.

## Mitochondria and stem cell fate regulation

Stem cells possess the capabilities of self-renewal and differentiation into multiple lineages. The balance between maintaining pluripotency and differentiating into tissue-specific cells relies on cell fate determination, where transcription factors, chromatin modifications, and extracellular stimuli play key roles in regulating stem cell fate and function. Recent research indicates that mitochondrial metabolism also significantly influences stem cell fate and function (9). Mitochondria play diverse roles in different cell types and are crucial in regulating cell fate. They are not only the center of energy metabolism but also participate in various important biological processes, including cell growth, differentiation, apoptosis, and stress responses. Mitochondria play a key role in maintaining, proliferating, and differentiating stem cells. Stem cells typically have lower mitochondrial activity, which helps maintain their self-renewal potential. In neural stem cells, mitochondrial dynamics (fusion and fission) regulate NSC proliferation and fate by coordinating nuclear transcription programs (35). Mitochondrial metabolism correlates with cell pluripotency, and blocking mitochondrial glycolysis can reduce reprogramming efficiency. iPSCs producing varying levels of ATP exhibit different degrees of pluripotency. Studies have constructed ESC models carrying mtDNA mutations that do not affect genomic DNA, demonstrating that mtDNA mutations can impair embryonic stem cell pluripotency by regulating metabolic activity (36). During the reprogramming of somatic cells such as fibroblasts into iPSCs, mitochondrial morphology, ultrastructure, and distribution undergo reversible changes. Additionally, metabolic processes in mitochondria shift, with stem cells primarily relying on glycolysis for energy and producing large amounts of lactate, while differentiated cells primarily generate energy through oxidative phosphorylation, resulting in decreased glycolytic rates, increased oxygen consumption, and higher ATP content (37). Inhibiting or promoting glycolysis can suppress or enhance somatic cell reprogramming. Recent studies found that mitochondrial fission or formation of “donut” shapes, secreted into the extracellular bone matrix, promotes osteoprogenitor cell differentiation and maturation, accelerating bone regeneration (38).

## Mitochondria and longevity

Recent advances in aging research suggest that interventions affecting multiple organs can extend healthspan and delay age-related diseases. These new interventions’ effects can be partially explained by mitochondrial physiology. Excess carbohydrates and calories weaken OXPHOS and antioxidant defenses, maintaining the electron transport chain (ETC) in a prolonged reduced state. This environment favors ROS production and mtDNA mutations, leading to premature cell death (39). Caloric restriction interventions reduce excess calorie intake, relieving the ETC from a reduced state, lowering ROS production, inducing OXPHOS, and enhancing antioxidant defenses, thus preventing age-related diseases and extending lifespan. There is evidence of reciprocal and multi-level interactions among longevity pathways, coupled with the complex regulation of mitochondrial physiology under caloric restriction, supporting the hypothesis that mitochondria may participate in lifespan regulation (13). Overall, this system is multifactorial, highly dynamic, and controls the aging process. Inhibiting the insulin/IGF-1 signaling (IIS) pathway significantly extends the lifespan of various organisms, suggesting it is an evolutionarily conserved mechanism (40). Extensive gene expression overlap was found between insulin receptor DAF-2 mutants and long-lived mitochondrial mutant strains in C. elegans, with DAF-2 mutants showing an overall reduction in mitochondrial protein turnover (41). Additionally, DAF-2 mutants participate in OXPHOS protein abundance, enhancing respiratory capacity and membrane potential. Increased respiratory capacity detected in DAF-2 mutants contrasts with observations in adult wild-type N2 worms (42). Similarly, FIRKO mice, with fat-specific insulin receptor gene knockout, have longer lifespans despite exhibiting a lean phenotype from an early age (3 months old) and normal food intake throughout their lives. Compared to age-matched control mice, FIRKO mice aged 2.5-3.0 years show increased gene expression for proteins involved in key metabolic processes such as glycolysis, TCA cycle, β-oxidation, and OXPHOS. Interestingly, when insulin receptors in adult mouse liver, fat, and muscle tissues are disrupted, the same phenotype is not observed, highlighting the complex role of the mammalian IIS pathway (43). In response to caloric restriction, yeast NAD biosynthesis gene PNC1 is induced, catalyzing the first and rate-limiting step of NAD+ biosynthesis. The resulting increase in NAD+ availability enhances SIR2 activity, promoting mitochondrial respiration and inhibiting DNA recombination, a known cause of yeast aging (44). Interestingly, increased expression of the mitochondrial biogenesis transcription driver HAP4 extends yeast lifespan under normal calorie conditions, and caloric restriction does not further extend this longevity benefit (33). In rodents, age-dependent NAD+ level decline reduces SIRT1 activity, ultimately affecting mitochondrial homeostasis (45). Interestingly, SIRT3 is considered a mediator of some caloric restriction’s longevity benefits by targeting and deacetylating mitochondrial proteins.

## The role of mitochondria in aging, cell death, tumor immunity

Mitochondria play a central role in cellular aging, cell death, tumor immunity, and cancer progression. Mitochondrial dysfunction leads to increased oxidative stress, metabolic reprogramming, and alterations in cell fate. In cellular aging, mitochondrial functional decline is a key driving factor; in cell death, mitochondria are critical regulators of apoptosis and pyroptosis. In tumor immunity, mitochondrial metabolites and dynamics influence immune cell activity and the tumor microenvironment. During cancer progression, mitochondria provide energy and metabolic intermediates that support tumor cell proliferation and invasion. These findings provide a crucial theoretical foundation for the development of mitochondria-targeted anticancer strategies. Herein, we systematically summarize the functions and mechanisms of mitochondria in cellular aging, cell death, tumor immunity, and cancer progression (**Table 1**).

**Table 1:** Summary the functions and mechanisms of mitochondria in different cell death, cellular aging, tumor immunity, and cancer progression.

Process	Function	Mechanism
Apoptosis	Release of cytochrome c from mitochondria	Mitochondria play a central role in apoptosis. The mitochondrial outer membrane becomes permeable, allowing the release of cytochrome c into the cytoplasm. Cytochrome c activates caspase-9 and caspase-3, triggering a cascade of proteolytic events that lead to DNA fragmentation, membrane blebbing, and ultimately cell death.
Necrosis	Mitochondrial dysfunction	Necrosis is often caused by external injury or internal stress (such as hypoxia or oxidative damage), leading to mitochondrial membrane rupture. This results in mitochondrial dysfunction, reduced ATP production, and subsequent membrane instability, ultimately causing the release of intracellular contents and triggering inflammation.
	Decreased ATP synthesis	
	Membrane rupture leading to leakage of cell contents	
Ferroptosis	Iron-dependent oxidative damage	Ferroptosis is an iron-dependent form of cell death where mitochondria play a crucial role. Excessive iron accumulation promotes the generation of reactive oxygen species (ROS), which damages mitochondrial membranes. This leads to lipid peroxidation, mitochondrial dysfunction, and eventual cell death. Mitochondrial iron homeostasis and oxidative stress response are pivotal in ferroptosis.
Cuproptosis	Copper ion accumulation	Cuproptosis is a newly discovered form of cell death induced by copper ion overload. Copper interacts with mitochondrial proteins, causing abnormal protein modifications, disrupting mitochondrial function. This inhibits mitochondrial respiration, leading to mitochondrial dysfunction and ultimately cell death. Copper-mediated mitochondrial protein stress plays a key role in cuproptosis.
	Mitochondrial protein modification	
	Inhibition of mitochondrial respiration chain	
Cellular Senescence	Mitochondrial dysfunction	Mitochondria are central to cellular senescence. As cells age, mitochondrial function declines, leading to an accumulation of oxidative stress. Senescent cells release pro-inflammatory cytokines, a phenomenon known as the senescence-associated secretory phenotype (SASP), which further influences the surrounding microenvironment and contributes to tissue aging.
	Increased oxidative stress	
	Secretion of pro-inflammatory cytokines (SASP)	
Tumor Immunity	Role of mitochondria in immune cell function	Mitochondria are crucial for the energy metabolism and function of immune cells such as T cells and natural killer (NK) cells. Tumor cells often reprogram mitochondrial metabolism to evade immune surveillance. Mitochondria also regulate immune signaling pathways, including calcium flux and ROS production, which influence immune responses.
	Metabolic reprogramming of immune cells	
Cancer Progression	Mitochondrial metabolic reprogramming	Mitochondria are essential for cancer progression by supporting tumor metabolism, cell proliferation, and survival. Cancer cells often exhibit altered mitochondrial function, such as enhanced oxidative phosphorylation, altered mitochondrial biogenesis, and increased production of ATP through aerobic glycolysis. These changes help sustain the rapid growth and metastatic potential of tumors.
	Mitochondria in tumor metabolism	
	Involvement in cell proliferation, migration, and metastasis	

## The role of mitochondria in aging

Mitochondria are crucial organelles within cells, characterized by their complex structure. During cellular aging, both the function and structure of mitochondria undergo significant changes. Mitochondrial DNA is particularly susceptible to damage, leading to gene mutations and functional impairments. Furthermore, the decline in mitochondrial function results in reduced oxidative phosphorylation efficiency and diminished ATP production, causing an inadequate cellular energy supply. Mitochondria also generate increased levels of reactive oxygen species (ROS), which elevate intracellular oxidative stress, damaging cell membranes, proteins, and DNA, thereby accelerating the aging process. As mitochondrial autophagy (mitophagy) diminishes, damaged mitochondria are not effectively cleared, leading to accumulated damage that further compromises cellular health. In summary, mitochondria play a critical role in the process of cellular aging, and maintaining their function is essential for delaying cellular senescence.

### Mitochondrial ROS and aging

Mitochondrial dysfunction is closely associated with aging, and the intracellular level of reactive oxygen species (ROS) is a key determinant of lifespan. Mitochondria, being the primary organelles producing ROS within cells, generate ROS that can attack various mitochondrial components, leading to increased mtDNA mutations and oxidative damage to associated respiratory chain enzymes (46). Damage to respiratory chain enzymes further promotes ROS production, resulting in mitochondrial dysfunction, cellular senescence, and declining organ function. Peroxidases play a vital role in cellular antioxidant defense (47). Compared to wild-type mice, knockout mice lacking catalase exhibit accelerated aging phenotypes, and overexpression of mitochondrial peroxidase extends the lifespan of mice by 18%. Reduced catalase expression accelerates ROS production and thereby speeds up aging (48). Overexpression of catalase can also mitigate the effects of cardiac aging in mice; aged mice display more mitochondrial oxidative damage in the heart, along with increased mtDNA deletions and mutation frequencies compared to young mice (49). During normal aging, the accumulation of damaged mitochondria significantly reduces mitophagy levels in mammals, leading to functional deficits in aging tissues and organs. Simulating decreased mitophagy by knocking out autophagy-related genes reveals that dysfunctional mitochondria gradually accumulate, and ROS levels rise significantly when autophagy is disrupted (50). Increasing mitophagy can extend lifespan, as demonstrated in fruit flies, where overexpression of Parkin enhances mitophagy levels and prolongs lifespan (33). Additionally, compounds like polyamine spermidine can promote mitophagy, thereby extending lifespan.

### mtDNA mutations and aging

mtDNA mutations also affect individual and cellular aging, with the accumulation of mtDNA mutations increasing with age (51). Significant accumulation of mtDNA point mutations is observed in aging colonic crypts. Mitochondrial DNA polymerase gamma (POLG), as a repair enzyme in the mtDNA synthesis process, can reduce mtDNA mutation rates. Mutations in POLG impair its proofreading ability, leading to increased mtDNA mutations (52). Mice with POLG mutations exhibit premature aging phenotypes, including shortened lifespan, hair loss, kyphosis, anemia, and reduced mobility. Mitochondrial dysfunction is a hallmark of cellular senescence (53). Recently, researchers have found that mitochondrial outer membrane permeabilization associated with cell death occurs in senescent cells (54). During cellular senescence, a subset of mitochondria undergo outer membrane permeabilization, leading to the release of mtDNA into the cytoplasm through BAX and BAK pores, activating the cGAS-STING signaling pathway and promoting the senescence-associated secretory phenotype (55).

### Genomic instability and aging

Genomic instability in the nucleus can influence mitochondrial physiology during aging through so-called anterograde stimulation (56). Besides its key roles in organismal health and mitochondrial physiology, NAD+ serves as a rate-limiting metabolite for poly (ADP-ribose) polymerases (PARPs), which are critical DDR proteins (57). When DNA breaks occur, PARP1 senses the break and initiates the DNA repair signal. Activated PARP1 consumes NAD+ and generates PAR through a process called PARylation (58). Additionally, studies in animal models and cells from xeroderma pigmentosum and Cockayne syndrome patients show that DNA damage-induced SIRT1 activity reduction and persistent PARP activation lead to mitochondrial imbalance (59). Pharmacological supplementation of NAD+ or inhibition of PARP1 can restore SIRT1 activity and mitochondrial homeostasis. Research on ATM supplements the link between PARP1 and mitochondrial physiology. ATM, caused by defects in the ATM gene encoding a DNA repair protein recruited by PARP1 to DNA break sites, is associated with mitochondrial dysfunction, increased ROS levels, and the accumulation of damaged mitochondria. Similarly, Atm-/- mice exhibit mitochondrial defects, and NAD+ supplementation-based interventions mitigate their health decline (60).

## The role of mitochondria in cell death

**Figure 4:**
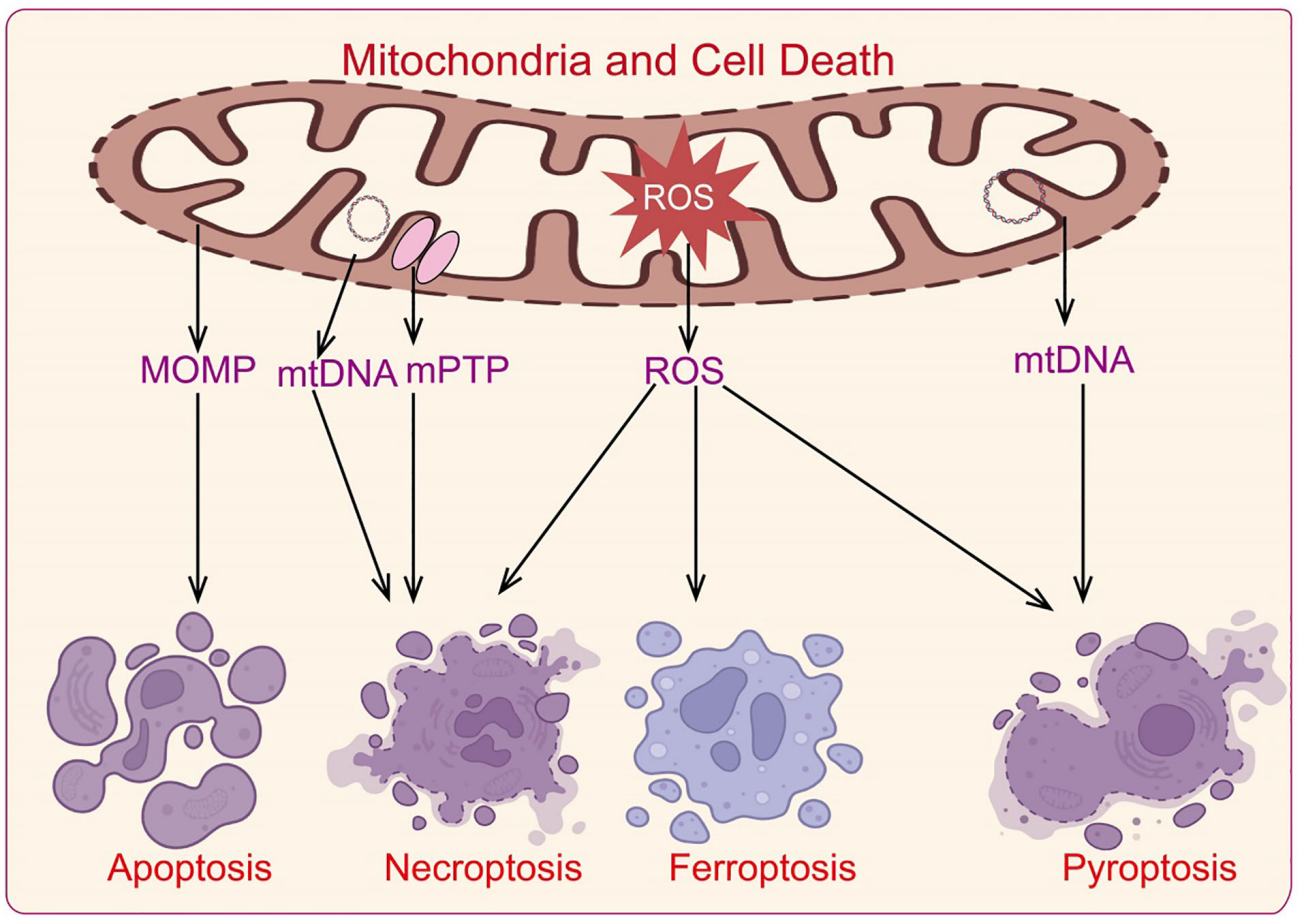
Mechanisms by which mitochondria regulate cell death.

Cell death is a crucial regulatory mechanism for maintaining organismal homeostasis. Mitochondria are involved in classic apoptosis by regulating cytochrome c release and caspase family protein activation (61). They also participate in various forms of cell death, including necrosis, pyroptosis, and ferroptosis (62). Abnormal regulation of cell death is a significant cause of neurodegenerative diseases and autoimmune disorders, with mitochondria playing a role in the development and progression of various diseases through their regulation of cell death mechanisms (**Figure 4**).

### Apoptosis

Apoptosis is a programmed cell death mechanism orchestrated by genetic regulation to maintain internal homeostasis. This process ultimately results in the clearance of apoptotic cells by macrophages. Apoptosis can be mediated via two main pathways: the intrinsic (mitochondrial) pathway and the extrinsic (death receptor) pathway (63). The intrinsic pathway involves mitochondrial mediation and endoplasmic reticulum (ER)-mediated signaling.

The mitochondrial apoptotic pathway is triggered by intracellular stimuli such as DNA damage, growth factor deprivation, and mitotic arrest. These stimuli lead to the activation of the B-cell lymphoma 2 (BCL-2) protein family, which includes both anti-apoptotic proteins (e.g., BCL-2) and pro-apoptotic proteins, such as BH3-only members (Bid, Bik, Bad, Bim, etc.) and effector proteins like Bax and Bak (64). Bax and Bak increase outer mitochondrial membrane permeability, leading to the release of pro-apoptotic factors (e.g., cytochrome c, apoptosis-inducing factor (AIF), endonuclease G (ENDOG), SMAC/DIABLO, HTRA2/OMI) into the cytosol. Cytochrome c then interacts with apoptosome components in the presence of ATP and dATP, forming the apoptosome complex, which recruits and activates caspase-9. Caspase-9 subsequently activates caspase-3 and caspase-7, initiating the caspase cascade that drives apoptosis (65). The release of mitochondrial cytokines also contributes to apoptosis: AIF and ENDOG translocate to the nucleus causing chromatin condensation and fragmentation, while SMAC/DIABLO and HTRA2/OMI neutralize anti-apoptotic proteins such as XIAP, further promoting apoptosis (66).

### Necroptosis

Necroptosis, a form of programmed cell death resembling necrosis, occurs when apoptosis is inhibited. This process features cellular swelling, membrane rupture, and the release of damage-associated molecular patterns (DAMPs), pro-inflammatory cytokines, and chemokines (2). Necroptosis primarily involves the phosphorylation and activation of mixed lineage kinase domain-like pseudokinase (MLKL) (67). Research shows that when caspase-8 is inhibited, tumor necrosis factor-alpha (TNFα) binds to death receptors on the cell membrane (e.g., TNFR1, Fas/CD95), activating these receptors and binding with adaptor proteins TRADD and TRAF2 (68). This interaction mediates the translocation of receptor-interacting protein kinase 1 (RIPK1) to the TNF receptor, forming a necrosome complex with RIPK3 and phosphorylating MLKL. Activated MLKL translocates to the plasma membrane, causing increased membrane permeability through interaction with phosphatidylinositol, leading to Ca^2+^ influx and cellular destruction (69). Additionally, mitochondrial reactive oxygen species (ROS) participate in a positive feedback loop in necroptosis, increasing RIPK1 serine autophosphorylation, thus promoting necroptosis. RIPK1 and RIPK3 also enhance mitochondrial metabolism, increasing mitochondrial ROS levels and stabilizing the necroptotic complex. RIPK3 can further activate pyruvate dehydrogenase complex through phosphorylation, promoting aerobic respiration and ROS production, thereby facilitating necroptosis (70).

### Pyroptosis

Pyroptosis is a form of programmed cell death induced by inflammasomes, primarily in response to pathogen invasion and oxidative stress (71). Unlike apoptosis and necroptosis, pyroptosis is characterized by continuous cell swelling until membrane rupture and subsequent release of intracellular contents, triggering a strong inflammatory response (72). Pyroptosis depends on the activation of caspase family proteins and Gasdermin (GSDM) proteins. Upon detection of pathogen-associated molecular patterns, inflammasomes are activated and recruit pro-caspase-1. Activated caspase-1 cleaves GSDMD, releasing its N-terminal domain, which increases the permeability of the plasma membrane and mitochondrial outer membrane (73). This results in the release of pro-inflammatory cytokines such as IL-1β and IL-18 and changes in osmotic pressure, leading to cell swelling, rupture, and pyroptosis. Other caspase proteins, including caspase-4 and caspase-5, interact directly with intracellular lipopolysaccharides to regulate IL-1β and IL-18 maturation and release, or directly cleave GSDMD, inducing NLRP3 inflammasome assembly and promoting pyroptosis. Additionally, NLRP3 activation requires Ca^2+^ signaling, with Ca^2+^ overload causing mitochondrial damage and ROS release, which activates NLRP3 (2). Inflammasomes also increase mitochondrial outer membrane permeability by cleaving and activating BH3-only protein BID, leading to downstream caspase-3 activation, potassium channel opening, and potassium release, thus promoting inflammasome assembly.

### Ferroptosis

Ferroptosis is a form of cell death driven by the accumulation of lipid peroxides dependent on iron ions (74). Cells combat ferroptosis through endogenous glutathione peroxidase 4 (GPX4) activity, which reduces lipid peroxides to lipid alcohols, with glutathione (GSH) being oxidized in the process. GPX4 knockout directly induces lipid peroxidation in mouse cells, leading to ferroptosis. GSH, as a cofactor for GPX4, requires cysteine for its synthesis. Cysteine enters cells via the cysteine/glutamate antiporter System Xc^-^, composed of SLC7A11 and SLC3A2. SLC7A11 specifically uptakes extracellular cysteine and releases glutamate, with increased SLC7A11 expression promoting GSH synthesis and enhancing resistance to ferroptosis (75). Thus, extracellular glutamate concentrations can inhibit System Xc^-^ and induce ferroptosis. SLC7A11 is also regulated by endogenous factors such as the tumor suppressor gene p53, which downregulates SLC7A11 expression, decreasing cysteine uptake, reducing GPX4 activity, and impairing ferroptosis resistance (76). Iron ions are crucial for ferroptosis, and mitochondria, as key organelles for iron utilization, catalysis, and metabolism, play a central role in iron homeostasis and ferroptosis (77). Under pathological or stress conditions, excessive ROS generated from oxidative phosphorylation (OXPHOS) reacts with ferrous iron, promoting lipid peroxidation and disrupting redox balance. While mitochondria do not play a role in GPX4-induced ferroptosis, they are critical in cysteine deficiency-induced ferroptosis (78). Cysteine deficiency leads to lipid peroxide accumulation, glutamine breakdown, accelerated TCA cycle, increased mitochondrial respiration, hyperpolarization, ROS production, and ultimately ferroptosis (79).

## The role of mitochondria in tumor immunity

**Figure 5:**
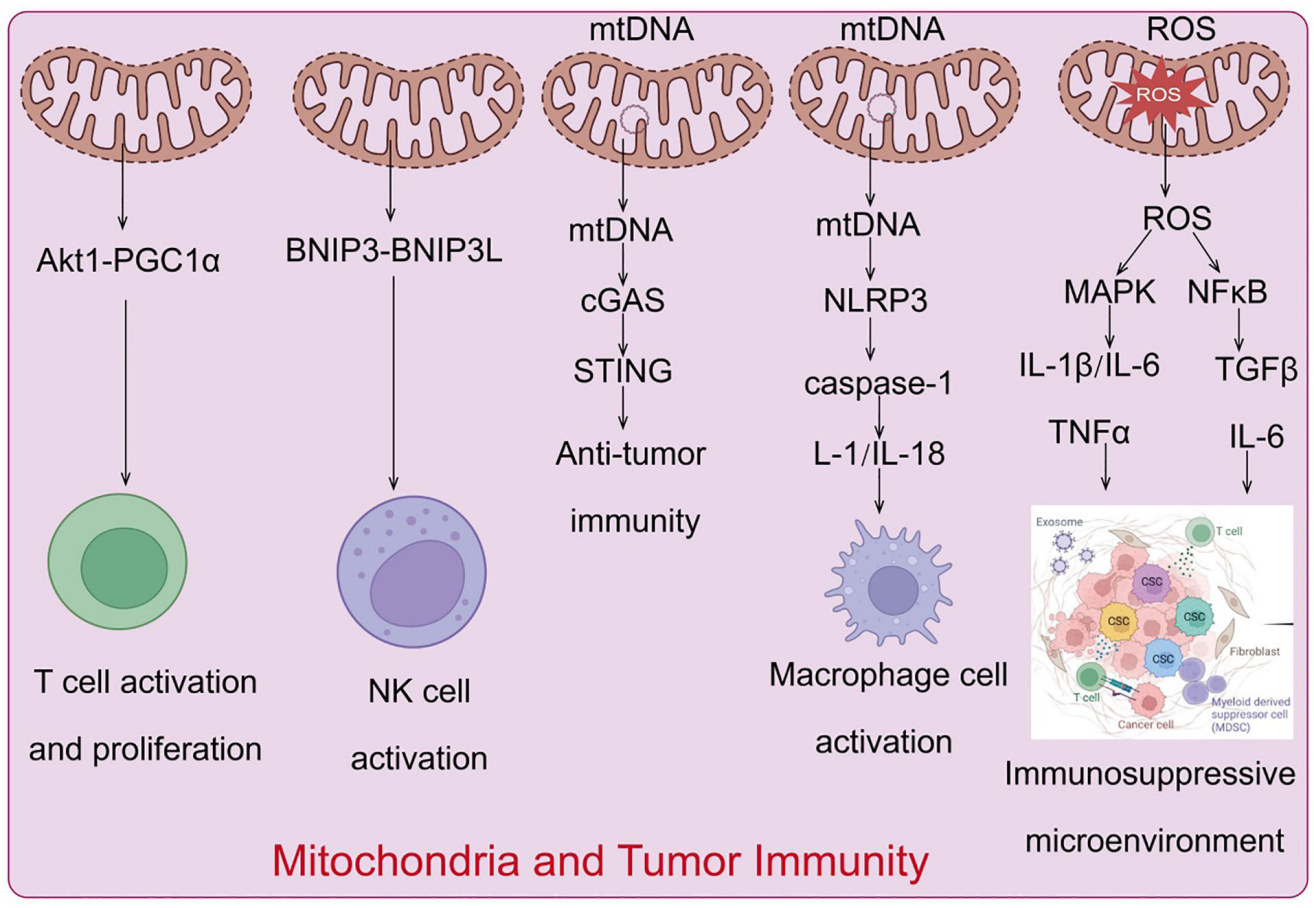
Mechanisms by which mitochondria regulate the tumor immune microenvironment.

Mitochondria evolved from prokaryotic α-proteobacteria, are diverse organelles found in most eukaryotic cells. Mitochondria are the only organelles in animal cells, apart from the nucleus, that contain DNA. Mitochondrial DNA (mtDNA) is a circular molecule encoding 13 proteins essential for oxidative phosphorylation complex formation, 22 transfer RNAs, and 2 ribosomal RNAs necessary for mitochondrial translation (80). As the center of cellular energy metabolism and biosynthesis, mitochondria produce ATP through oxidative phosphorylation to maintain cellular energy homeostasis (81), while metabolic intermediates play crucial roles in biosynthetic pathways. Furthermore, mitochondria regulate various cellular processes, including energy metabolism, cell fate determination, and immune responses, by releasing mtDNA, mtROS, and metabolic products. Consequently, numerous studies indicate that mitochondrial dysfunction is associated with various diseases, including autoimmune diseases (82), and that dysfunctional mitochondria drive cellular malignant transformation (83). Tumor cells reprogram metabolic pathways to meet the increasing demands for energy and biosynthesis. In the tumor microenvironment (TME), nutrient depletion and excessive production of metabolic byproducts driven by tumor development regulate metabolic reprogramming and signal activation in tumor-infiltrating immune cells, controlling the polarization of different immune cell types. This induces metabolic disorder-mediated antitumor immune response deficiency, establishing an immunosuppressive TME. As highly dynamic organelles with diverse biological functions (84), mitochondria play a key regulatory role in metabolic regulation and immune cell activation. Studies have shown that mitochondrial dysfunction in various cells, including tumor and immune cells in the TME, is a major cause of cancer development, progression, and metastasis (85) (**Figure 5**).

### Mitochondrial regulation of immune cell activation, proliferation, and differentiation

The mitochondria of naive T cells are typically fragmented and spherical, with oxidative phosphorylation and fatty acid oxidation being the main pathways to meet their energy needs in a quiescent state (86). Upon T cell activation, there is a significant increase in the activity of aerobic glycolysis and fatty acid synthesis-related signals, supporting their proliferation and differentiation capabilities and promoting their biological functions (87). Additionally, mitochondria gather beneath the T cell receptor clusters The activation of T cell receptors enhances calcineurin activity, which activates dynamin-related protein 1 (88), inducing further mitochondrial fission and cristae relaxation. Increased mitochondrial number and cristae relaxation play important regulatory roles in glycolysis-related signaling during effector T-cell activation (21). Mitochondria also play crucial roles in T-cell signal transduction and cell fate determination. During T cell activation, mitochondria accumulate at the immunological synapse formed between T cells and antigen-presenting cells (89). T-cell receptor activation stimulates the production of reactive oxygen species (ROS) and ATP, essential for maintaining calcium ion homeostasis and activating downstream-related signals (90). When activated T cells transition into memory T cells or regulatory T cells, mitochondria gradually fuse into elongated structures, cristae become tighter, and the metabolic state shifts to fatty acid oxidation and oxidative phosphorylation, maintaining cell phenotype, survival, and functional transformation (91). In tumor tissues, tumor cells outcompete immune cells for glucose and other nutrients, inhibiting immune cell metabolism and function (92). Tumor-infiltrating T cells in the TME experience chronic oxidative stress, and their proliferation capacity, membrane integrity, and related signaling pathways are severely affected due to metabolic insufficiency induced by glucose and oxygen deprivation and persistent mitochondrial dysfunction mediated by the Akt1-PGC1α signaling pathway (75). This severely disrupts T cell antitumor immune effects and cytokine production, ultimately leading to immunosuppression and tumor immune evasion.

### Mitochondrial regulation of NK cell activation

Natural killer (NK) cells are innate cytotoxic lymphocytes whose activity is significantly correlated with glucose metabolism levels. Elevated glucose levels enhance NK cell activity (93). Activated NK cells exhibit significantly increased glycolytic capacity, basal oxidative phosphorylation rate, and maximal respiration capacity. During the transition to memory stages, mitochondrial autophagy proteins BNIP3 and BNIP3L promote mitochondrial autophagy, removing damaged mitochondria and reducing ROS production, facilitating the formation of memory NK cells (94). Studies have shown that in the hypoxic TME, the mitochondrial morphology of tumor-infiltrating NK cells is notably fragmented compared to normal NK cells, resulting in significantly reduced NK cell activity and tumor-killing capacity, leading to the loss of tumor immune surveillance ability (95).

### Mitochondria in macrophage polarization

Mitochondria play a key role in macrophage polarization (96). Macrophages can be divided into two subtypes: M1 pro-inflammatory macrophages activated by lipopolysaccharides/γ-interferon and M2 anti-inflammatory macrophages activated by IL-4. During polarization to the M1 subtype, cellular metabolism shifts from oxidative phosphorylation to aerobic glycolysis, increasing intracellular ROS levels, accompanied by increased mitochondrial fission. In contrast, during polarization to the M2 subtype, oxidative phosphorylation and fatty acid oxidation levels significantly rise, with mitochondria exhibiting a fused elongated state (97, 98).

### Mitochondrial DNA-mediated activation of innate immune signaling and regulation of antitumor immunity

While numerous studies have highlighted the significance of mitochondrial DNA (mtDNA) in the activation of innate immunity, the precise mechanisms governing mtDNA translocation from the mitochondrial matrix to the cytosol remain unclear. Independent studies in 2014 demonstrated that mtDNA is released during mitochondrial apoptosis (99), and subsequent research indicated that apoptosis-associated mitochondrial outer membrane permeabilization plays a crucial regulatory role in mtDNA release (100). Moreover, voltage-dependent anion channels, which facilitate the transport of metabolites and ions, can form oligomers in the mitochondrial outer membrane. Short mtDNA fragments translocate through these oligomers into the cytosol, triggering type I interferon signaling; additionally, cell stress-induced formation of mitochondrial permeability transition pores contributes to the release of short mtDNA fragments (101). The release of mtDNA in tumor immunity has garnered significant attention. Under normal conditions, mitochondrial DNA is tightly protected to prevent its release into the cytoplasm. However, in tumor cells, mitochondrial dysfunction, oxidative stress, and other factors may lead to mtDNA leakage. Released mtDNA acts as a danger-associated molecular pattern (DAMP) and regulates tumor immune responses through various pathways (102). Released mtDNA can activate the intracellular cGAS-STING pathway, which detects foreign DNA and triggers a downstream type I interferon response, thereby initiating innate immune responses (103). Additionally, mtDNA can activate the NLRP3 inflammasome, promoting the secretion of pro-inflammatory cytokines such as IL-1β and IL-18, further driving inflammatory responses. These signaling pathways not only enhance anti-tumor immune responses but may also trigger immune evasion mechanisms, promoting tumor growth and metastasis.

When abnormalities in mtDNA integrity, replication, and damage repair lead to mtDNA leakage, the cytosolic DNA sensor cGAS rapidly detects mtDNA and induces the production of the second messenger 2’3’-cGAMP. Subsequently, 2’3’-cGAMP activates the endoplasmic reticulum-located adaptor protein STING, mediating the activation of downstream type I interferon signaling pathways and associated inflammatory responses (104). Recent research has revealed that cGAS-STING signaling activation plays a pivotal regulatory role in antitumor immunity. Tumor T cell infiltration is positively correlated with overall prognosis in various cancer types (105). Tumor-specific adaptive immune responses, including the activation of cytotoxic T cells (CD8+ T cells), rely on type I interferon signaling from antigen-presenting cells, with the cGAS-STING pathway being a critical mediator of type I interferon activation (106). Activating cGAS-STING signaling with small molecule drugs can trigger relevant innate immune signals, activating various immune cells, including dendritic cells, macrophages, NK cells, and CD4+ and CD8+ T cells, resulting in significant tumor shrinkage or even complete regression *in vivo* (107). Studies have shown that intratumoral injection of 2’3’-cGAMP to activate STING stimulates the infiltration of activated CD8+ T cells and promotes tumor clearance in multiple subcutaneous tumor models, including colorectal cancer and melanoma (108). In summary, STING-dependent interferon regulatory factor 3 activation plays a critical role in connecting innate and adaptive immunity in CD8+ T cell-mediated antitumor responses. However, some reports indicate that STING activation may also promote tumor growth in certain cell types (109). Tumor cells can inhibit downstream type I interferon and canonical NF-κB signaling activation, promoting STING-dependent non-canonical NF-κB signaling, and enhancing tumor cell metastasis. For example, chromosomal instability activates STING-dependent non-canonical NF-κB signaling, promoting epithelial-mesenchymal transition, cell invasion, and metastasis (110). In models of breast cancer and lung cancer brain metastasis, tumor cells transfer 2’3’-cGAMP to astrocytes through tumor cell-astrocyte gap junctions, activating STING signaling in astrocytes, promoting the production of inflammatory cytokines, and subsequently activating STAT1 and NF-κB signaling in brain metastatic cancer cells, supporting tumor cell growth and chemotherapy resistance (111).

TLR9 recognizes the CpG domains of mtDNA, activating downstream MAPK and NF-κB signaling, promoting associated inflammatory responses. Notably, extracellularly leaked mtDNA can also activate TLR9 and cGAS-STING signaling in adjacent immune cells, regulating the polarization and function of various immune cells, including macrophages, dendritic cells, and T lymphocytes (112). As a cytosolic multiprotein complex, NLRP3 can recognize mtDNA leaked into the cytosol, activating downstream caspase-1, promoting the cleavage and activation of IL-1 and IL-18 precursors, recruiting macrophages, neutrophils, and T lymphocytes to participate in related immune responses (113). Simultaneously, NLRP3 and caspase activation can further mediate mitochondrial damage and promote mtDNA leakage (114). During tumor-induced mitochondrial stress, mtDNA is released into the cytosol and extracellular space, mediating multiple innate immune signaling activations, and significantly driving tumor development. For example, in primary hepatocellular carcinoma, dynamin-related protein 1 overexpression-mediated mitochondrial dysfunction can activate TLR9-NF-κB signaling through mtDNA release, enhancing chemokine ligand 2 secretion from tumor cells, promoting TAM recruitment and polarization, and driving cancer progression (115). In esophageal squamous cell carcinoma, dynamin-related protein 1 overexpression induces mitochondrial dysfunction and cytosolic mtDNA stress, subsequently inducing autophagy through cGAS-STING signaling activation, driving cancer progression (116). It is noteworthy that reactive oxygen species (ROS) stress can induce the generation of extracellular vesicles containing mtDNA and PD-L1, regulating the microenvironment surrounding the tumor tissue (117). These extracellular vesicles can subsequently inhibit T cell immunity in the tumor microenvironment by promoting macrophage secretion of interferon and IL-6. Recent reports indicate that elevated exosomal PD-L1 levels in various cancer patients are positively correlated with mtDNA and interferon-γ production (118). In conclusion, multiple studies suggest that mtDNA-containing extracellular vesicles are key components influencing metabolism and promoting tumor growth, and the MDV-dependent mitochondrial quality control system is crucial for cell survival and inflammatory characteristics (119). In summary, the release of mtDNA is not only a response mechanism of tumor cells to internal and external stressors but also a crucial regulatory factor in tumor immune evasion. Understanding mtDNA release and the associated signaling pathways is of significant importance for developing new immunotherapeutic strategies and improving cancer treatment outcomes.

## Critical regulatory role of mitochondrial reactive oxygen species in tumor immune evasion

Cellular oxidative stress refers to the process where cells experience harmful stimuli or significant metabolic changes, leading to the excessive production of highly reactive molecules like reactive oxygen species (ROS), causing an imbalance between oxidation and antioxidation systems and resulting in cellular damage (120). Mitochondria are the primary cellular organelles generating ROS through aerobic respiration’s electron transport chain and oxidative phosphorylation (121). Under physiological conditions, low levels of ROS act as crucial signaling molecules in cellular signal transduction, regulating gene expression, cell proliferation, differentiation, and stress responses. However, excessively high ROS levels cause oxidative damage to nuclear and mitochondrial DNA, proteins, and lipids, ultimately leading to cellular injury. Therefore, maintaining a dynamic balance between ROS production and consumption is vital for cellular homeostasis and overall health (122).

Compared to normal cells, tumor cells often harbor higher ROS levels. Multiple pro-tumor events, including oncogene activation, loss of tumor suppressor function, mitochondrial activity changes, and tissue inflammation, lead to excessive ROS production, while ROS-mediated oxidative stress further drives the pathological processes of inflammation, fibrosis, and tumors (123). As a crucial mediator in tumor development, ROS plays significant regulatory roles in various aspects of tumor cell proliferation, migration, invasion, angiogenesis, inflammation, and immune evasion. It helps tumor cells adapt to harsh survival environments, and its mediated inflammatory responses can alter the immune cell composition within the tumor microenvironment, influencing its immunosuppressive nature (124). It is important to note that ROS is a double-edged sword in tumor development; chemotherapy and radiotherapy may induce substantial ROS production in tumor cells, promoting tumor cell death and increasing sensitivity to antitumor treatments.

## ROS promotes the formation of an immunosuppressive tumor microenvironment

Current research widely acknowledges that the tumor microenvironment is a chronic inflammatory environment, with ROS playing a central regulatory role in its formation, ultimately driving cancer development (125). Tumor cells adapt to high ROS environments by inducing inflammatory cytokine secretion, stabilizing hypoxia-inducible factor-1α, activating AMPK signaling, and promoting NADPH production, thereby avoiding cell death, promoting tumor metastasis, and angiogenesis (126). ROS can induce MAPK signaling activation, regulating the secretion of NF-κB-mediated inflammatory cytokines such as IL-1β, IL-6, and tumor necrosis factor-α, thereby modulating tumor cell inflammatory responses (127). Additionally, ROS regulates the activation states of immune cells within the tumor microenvironment that determine cancer progression. High ROS levels inhibit T cell receptor-antigen peptide-MHC complex formation on infiltrating T cells within the microenvironment, suppressing T lymphocyte activation and enabling tumor cells to evade immune system attacks, thereby promoting cancer progression (128). Studies have also found that tumor cells and immunosuppressive cells within the microenvironment synergistically induce mtROS production, aiding tumor tissues in establishing immune tolerance (129).

## The role of mitochondria in cancer

Mitochondria play a dual role in cancer progression, acting both as promoters and suppressors depending on the cellular context. On one hand, mitochondria are central to energy production and biosynthesis, supporting rapid cell proliferation in cancer. They contribute to tumor progression by enhancing oxidative phosphorylation (OXPHOS), producing metabolites that fuel the tricarboxylic acid (TCA) cycle, and generating reactive oxygen species (ROS), which can promote genetic instability and oncogenic signaling. On the other hand, mitochondrial dysfunction can act as a barrier to tumorigenesis. For instance, excessive ROS levels can induce oxidative damage and apoptosis, while mutations in mitochondrial DNA (mtDNA) or disruptions in mitochondrial metabolism can impair tumor growth by limiting energy supply or triggering metabolic stress. This duality highlights mitochondria as potential therapeutic targets, where strategies could either inhibit their pro-tumorigenic functions or exploit their vulnerabilities to suppress cancer development.

### Mitochondrial promotes tumor progression

**Figure 6:**
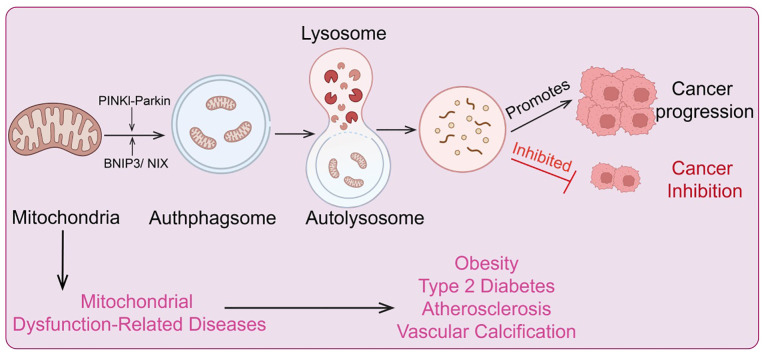
Regulatory mechanisms of mitochondrial metabolism and autophagy, and diseases associated with metabolic and autophagic dysregulation.

Mitochondrial autophagy can prevent cancer cells from being induced into differentiation and programmed cell death by sodium butyrate, and it allows for the digestion and utilization of damaged mitochondria, thereby promoting tumor cell survival (130). Studies have shown that hypoxia can enhance autophagy in colon cancer cells HCT116 by regulating translation levels. During this process, highly conserved lysosomal glycoproteins PSAP and LAMP2 induce mitochondrial autophagy through upregulation of translation levels, thus protecting tumor cells (131). Furthermore, 12 mitochondrial-specific glycoproteins, such as MRPL36, can also promote mitochondrial autophagy in colon cancer cells by increasing translation levels, which supports the survival of HCT116 cells (132). BNIP3 and NIX are two crucial proteins mediating mitochondrial autophagy and play roles in tumor development. Under hypoxic conditions, BNIP3 and NIX are highly expressed in breast cancer, endothelial cancer, and epithelial cell carcinoma compared to healthy individuals. Additionally, BNIP3 is also highly expressed in lung cancer and follicular lymphoma (133). However, in primary colon cancer, methylated BNIP3 does not exhibit high expression but rather shows a downregulated trend, and this downregulation is associated with abnormal methylation mediated by DNA methyltransferase 3β (DNMT3β) and DNA methyltransferase 1 (DNMT1) (134). The downregulation of BNIP3 accelerates colon cancer cell growth and reduces sensitivity to chemotherapy, potentially related to BNIP3’s cellular localization. For instance, in glioblastoma tumor cells, despite increased BNIP3 expression in hypoxic regions, its localization is not in mitochondria or the cytoplasm but in the nucleus. Current evidence suggests that its nuclear localization is a marker of tumor dormancy, though its other potential functions within the nucleus remain unclear (135) (**Figure 6**).

Other factors regulating hypoxia-induced mitochondrial autophagy, such as Src and CK2, also play significant roles in cancer cell homeostasis and tumor progression. Both factors have carcinogenic properties and are crucial participants in cancer development (136). Additionally, CNX is another component associated with hypoxia-induced mitochondrial autophagy and is highly expressed in cancer cells. This feature makes CNX a significant prognostic marker for tumors (137). Research has shown that CAV1 in the extracellular matrix is a potent biomarker for tumor progression and metastasis. ROS released by cancer cells can induce the loss of matrix CAV1, leading to metabolic changes in tumor matrix cells, mitochondrial dysfunction, and further enhancement of mitochondrial autophagy (138). Notably, studies on miRNAs in CAV1-deficient tumor matrix cells revealed two key cancer-related miRNAs, MIR31 and MIR34C, which drive mitochondrial autophagy. This function is closely linked to their ability to induce mitochondrial oxidative stress and activate hypoxic responses/HIF1a (139). This finding provides new insights into the role of miRNA regulation in mitochondrial autophagy during cancer development. Furthermore, CAV1 can negatively regulate transforming growth factor β (TGFβ) signaling, and TGFβ signaling activation is crucial for inducing mitochondrial autophagy in tumor matrix cells. In fact, in tumor matrix cells, TGFβ signaling activation, either through paracrine or autocrine mechanisms, induces metabolic abnormalities in the tumor microenvironment (140). As a well-known tumor suppressor gene, BRCA1 usually inhibits tumor growth by maintaining genomic integrity. However, BRCA1-deficient cancer cells can influence the metabolism of surrounding fibroblasts by producing large amounts of hydrogen peroxide, which activates nuclear factor-kappa B (NF-κB) signaling to induce mitochondrial autophagy in tumor matrix cells, thereby promoting tumor growth (141). Besides ROS, other cytokines or bioactive factors, such as migration-stimulating factors and genetic truncations of the N-terminal isoform of fibronectin, can also induce mitochondrial autophagy in tumor matrix cells through TGFβ and CD42-NF-κB activation, further promoting tumor cell growth (142). Overall, mitochondrial autophagy can be activated through carcinogenic signaling pathways (mainly TGFβ and NF-κB pathways), promoting tumor cell growth by regulating cancer cell metabolism. Thus, neutralizing ROS or inhibiting metabolic uncoupling to suppress TGFβ and NF-κB signaling levels in tumor cells and their surrounding microenvironment, and further suppressing mitochondrial autophagy in the tumor matrix, may be a viable strategy for inhibiting tumor cell growth (143). This provides valuable insights and potential anti-cancer strategies targeting mitochondrial autophagy.

### Mitochondrial autophagy and tumor suppression

Mitochondrial dysfunction can compromise cellular integrity and impair cellular function. Mitochondrial autophagy plays a crucial role in eliminating damaged mitochondria, facilitating mitochondrial renewal, and participating in the adaptation to stress by controlling mitochondrial quality (144). However, the mechanisms by which cells distinguish between functional and non-functional mitochondria remain incompletely understood (9). Dysregulation of mitochondrial autophagy is associated with the development of various diseases, including neurodegenerative disorders, type 2 diabetes, and cancer (145). Increasing evidence indicates that mitochondrial autophagy dysfunction is a major contributor to tumorigenesis. The PINK1/Parkin pathway is considered a primary mechanism of mitochondrial autophagy (146). PINK1, a serine/threonine kinase, contains a mitochondrial targeting sequence (MTS) at its N-terminus. Under normal conditions, PINK1 is translocated to the mitochondrial inner membrane, where it is cleaved by the mitochondrial protease PARL into a 52 kDa protein and subsequently further degraded by other mitochondrial proteases (147). Parkin is recruited by PINK1 and phosphorylated at serine 65 within PINK1’s ubiquitin-like domain, which enhances Parkin’s E3 ligase activity. This leads to the polyubiquitination of Parkin, which in turn recruits the p62/SQSTM1 adaptor protein and interacts with LC3, promoting autophagy (148). Consequently, loss of Parkin function inhibits mitochondrial autophagy, leading to ROS accumulation, which in turn affects the function of cells, tissues, organs, and even entire systems. Mutations in Parkin are commonly detected in various cancers, including lung cancer, glioblastoma, and colon cancer. The PARK2 gene, which encodes the Parkin protein, is located on human chromosome 6 (FRA6E, 6q26), a region highly susceptible to gene mutations (149). Research has shown that PARK2 is significantly associated with human colorectal adenomatous polyposis, and overexpression of PARK2 can inhibit colon cancer cell proliferation (150). Furthermore, crossbreeding PARK2 gene knockout mice with mice exhibiting colorectal adenomatous polyposis leads to a dramatic acceleration in intestinal adenoma development in the offspring, accompanied by increased polyp diversity, indicating that PARK2 functions as a tumor suppressor gene (151). Hypoxic conditions provide a favorable microenvironment for cancer stem cells, enhancing the likelihood of cancer progression and metastasis. Hypoxia activates hypoxia-inducible factor 1α (HIF1α), which increases the expression of BCL-2 and the adenoviral E1B 19 kDa interacting protein 3 (134). BNIP3, a pro-apoptotic protein, enhances mitochondrial autophagy by promoting the elimination of damaged mitochondria through inhibition of mitochondrial fusion (152). In malignant glioblastoma cells, BNIP3 expression increases under ceramide induction, further activating mitochondrial autophagy and leading to cancer cell death. Similarly, treatment with 3,4-dihydroxybenzyl alcohol in esophageal cancer cells activates mitochondrial autophagy due to high BNIP3 expression, ultimately resulting in cancer cell death (153). Another regulator of mitochondrial autophagy, FUN14 domain-containing protein 1 (FUNDC1), is a newly discovered mitochondrial autophagy receptor protein with significant roles in mediating mitochondrial autophagy. Under hypoxic conditions, protein phosphatase 5 dephosphorylates FUNDC1, promoting its interaction with LC3 and thus enhancing mitochondrial autophagy. In early-stage cervical cancer, FUNDC1 expression is significantly higher in cancer cells compared to adjacent normal cells (154). This high FUNDC1 expression is negatively correlated with tumor progression and patient prognosis and can induce cancer cell apoptosis and increase sensitivity to cisplatin and ionizing radiation. Moreover, targeting FUNDC1 with X-rays can enhance mitochondrial autophagy for targeted cancer therapy (155). Notably, DCT-1, a transcription factor homologous to the Caenorhabditis elegans NRF2, is upregulated under mitochondrial autophagy conditions and activated by SKN-1 during oxidative stress to maintain mitochondrial homeostasis (156). HIF1α can downregulate mitochondrial expression, whereas SKN-1 stimulates the expression of core mitochondrial components and promotes the assembly of new mitochondria. Kosztelnik et al. found that SKN-1, as a novel mitochondrial autophagy regulator, promotes mitochondrial autophagy regeneration (157). Another mitochondrial autophagy regulator, nuclear factor erythroid 2-related factor 2 (NRF2), is a key regulator of cellular redox homeostasis and also impacts mitochondrial function. Small molecule activators of NRF2 maintain mitochondrial function by promoting mitochondrial autophagy and counteracting oxidative stress-induced permeability transitions (158). These findings suggest that activation of SKN-1/NRF2/NFE2L2 can induce mitochondrial autophagy and mitochondrial biogenesis, providing a new target for cancer therapy. Additionally, these factors may jointly enhance mitochondrial autophagy and stability by counteracting the Warburg effect (a metabolic shift from oxidative phosphorylation to glycolysis in hypoxic solid tumors, leading to increased glucose uptake and decreased oxygen consumption) and thus protect mitochondrial metabolism from carcinogenic transformation (159). Another major protein linking mitochondrial autophagy and cancer is insulin/insulin-like growth factor (IGF). Insulin/IGF signaling plays a critical role in the development and progression of many human cancers. While its role in altering the tumor microenvironment is well established, its effects on other malignant tumor phenotypes, such as distant metastasis, are still under investigation (160). Downregulation of insulin signaling is a highly conserved evolutionary pathway that can extend mitochondrial lifespan. Specific targeting of insulin and IGF receptors in cancer cells is a promising anti-cancer strategy (161). Given that mitochondrial autophagy provides beneficial regulatory effects through low insulin signaling, increased insulin/IGF signaling can induce mitochondrial autophagy in certain types of tumors, thereby inhibiting tumor progression.

### Mitochondrial dysfunction-related diseases

Mitochondrial genetic metabolic abnormalities are closely associated with various diseases. Mitochondria serve as the central hub for cellular energy metabolism, responsible for generating ATP through oxidative phosphorylation. When mitochondrial DNA (mtDNA) undergoes mutations or incurs damage, it can lead to decreased efficiency of oxidative phosphorylation, resulting in reduced ATP production and subsequent cellular energy deficiency (162). Moreover, mitochondrial metabolic abnormalities can cause excessive production of reactive oxygen species (ROS), elevating intracellular oxidative stress, which damages cell membranes, proteins, and DNA. These changes are linked to the development of numerous degenerative diseases, cardiovascular diseases, and metabolic disorders (66). Understanding the relationship between mitochondrial genetic metabolic abnormalities and diseases is crucial for developing therapeutic strategies targeting these conditions (**Figure 6**).

#### Obesity

The World Health Organization defines obesity as an abnormal or excessive fat accumulation that may impair health. Excessive food intake and lack of exercise are the primary causes of obesity (163). In conditions of nutritional surplus, increased fatty acids are stored in large amounts of adipose tissue, leading to adipocyte hypertrophy and inadequate blood supply, which induces hypoxia. Hypoxia triggers macrophages in adipose tissue to secrete large amounts of pro-inflammatory factors, such as tumor necrosis factor-alpha (TNF-α). TNF-α induces adipocytes to release free fatty acids, which in turn bind to Toll-like receptors on macrophages and adipocytes, further activating inflammatory signaling pathways and promoting the release of inflammatory factors (164). Studies have shown that elevated levels of glucose and fatty acids directly impair mitochondrial function in adipocytes (165). Additionally, excessive activation of mineralocorticoid receptors during obesity can cause changes in mitochondrial function, including increased production of reactive oxygen species and uncoupling, leading to dysfunction of adipose tissue. Moderate weight loss can alleviate systemic inflammation and insulin resistance, improve mitochondrial dysfunction, and increase the expression of SIRT1 (Sirtuin1) and the antioxidant GPX1 (166). In the arcuate nucleus of the hypothalamus, neurons expressing agouti gene-related protein (AgRP) and proopiomelanocortin (POMC) regulate feeding behavior in opposing directions. High-fat diets increase AgRP neuron activity, which enhances food intake and disrupts systemic energy metabolism. However, the absence of Mfn2 in AgRP neurons can prevent adverse metabolic responses, reduce fat mass, restore insulin and blood glucose levels, and prevent obesity (167). Conversely, the effect in POMC neurons is entirely different, with Mfn2 deficiency causing severe obesity, binge eating, and endocrine disorders (17). Recent studies have also shown that high-fat diets lead to reduced mitochondrial volume, increased density, and elevated expression of the mitochondrial fission protein Drp1, which induces the expression of uncoupling protein 2 in hypothalamic microglia, increasing susceptibility to obesity (168). Normally, mitochondria clear dysfunctional mitochondria through autophagy. However, excessive mitochondrial autophagy reduces the number of mitochondria, leading to decreased substrate oxidation and exacerbating lipid accumulation (169). The role of mitochondrial autophagy in obesity requires further investigation.

Mitochondria usually clear reactive oxygen species through antioxidant systems, but impaired mitochondrial function in obese individuals can increase the production of these unstable molecules. Excessive eating, lack of exercise, and obesity can lead to long-term imbalances in energy metabolism, which may result in increased reactive oxygen species production. High-fat diets promote mitochondrial fatty acid β-oxidation, leading to excessive electron flow through cytochrome c oxidase and subsequent accumulation of reactive oxygen species (170) Reactive oxygen species play an important role in adipocyte formation, but during obesity, a vicious cycle of adipocyte hypertrophy → local increase in reactive oxygen species → adipocyte hyperplasia exacerbates obesity. These studies highlight the significant role of reactive oxygen species in the development and progression of obesity.

#### Type 2 diabetes

With increasing living standards, the prevalence of type 2 diabetes has surged, with insulin resistance and inadequate secretion being core factors in its pathogenesis (171). Mitochondria, as crucial regulators of insulin secretion, are closely linked to the onset of type 2 diabetes. Mitochondrial dynamics play a significant role in the development of type 2 diabetes. OPA1 is essential for the optimal function of the respiratory chain in β-cells (172). Research indicates that the loss of OPA1 leads to β-cell death, reduced insulin secretion, and impaired systemic glucose homeostasis. In type 2 diabetes patients, a reduction in skeletal muscle Mfn2 expression has been observed, with Mfn2 levels correlating positively with insulin sensitivity. Ramírez et al. found that during the transition from fasting to feeding, mice with Mfn1 deficiency in POMC neurons exhibit mitochondrial structural defects and loss of adaptive responses, leading to insufficient insulin secretion by pancreatic β-cells and abnormal glucose homeostasis (173). However, other studies have shown that Mfn1-deficient mice are protected from glucose intolerance and insulin resistance induced by a high-fat diet and are more sensitive to the hypoglycemic effects of metformin. Drp1 also contributes to type 2 diabetes by affecting insulin secretion and function. Hennings et al. found that Drp1 deficiency in pancreatic β-cells impairs insulin secretion (174). Conversely, Fealy et al. demonstrated that reduced Drp1 activity is significantly associated with improved fatty acid oxidation and insulin sensitivity and that exercise reduces Drp1 levels while increasing Mfn1 and Mfn2 expression (175). Additionally, metformin can activate AMPK to improve mitochondrial function and hyperglycemia in obese patients. These results suggest that the expression of mitochondrial dynamics-related proteins may underlie the development of type 2 diabetes, though their regulatory mechanisms require further investigation. Another potential mechanism underlying type 2 diabetes is the production of high levels of reactive oxygen species (ROS). Diabetic rats have been observed to have reduced mitochondrial ATP production and elevated ROS levels (176). Pancreatic β-cells are particularly susceptible to ROS and oxidative stress, with their antioxidant enzyme levels being notably lower compared to other metabolic tissues. Thus, high levels of ROS are considered a primary cause of β-cell dysfunction in diabetes. Excessive ROS production in mitochondria can activate inflammatory pathways, leading to impaired insulin signaling and insulin resistance. Houstis et al. found that ROS levels are elevated in insulin-resistant cell models induced by glucocorticoids or TNF-α, suggesting that increased ROS levels are a crucial trigger for insulin resistance. Overexpression of catalase in mitochondria can prevent insulin resistance (177). These seemingly contradictory results indicate that further exploration is needed to clarify the relationship between mitochondrial-derived oxidative stress and type 2 diabetes.

#### Atherosclerosis

Atherosclerosis forms the pathological basis of many cardiovascular diseases, beginning with endothelial damage that allows lipids to enter the subendothelial space. Subsequently, mononuclear macrophages and smooth muscle cells engulf these lipids, forming foam cells, and the accumulation of foam cells leads to the formation of fatty streaks (178). This process is followed by the formation of fibrous plaques and atherosclerotic plaques. Mitochondrial ROS can promote the oxidation of low-density lipoprotein (LDL) and proteins, causing damage to nuclear DNA and mitochondrial DNA in smooth muscle and endothelial cells. This damage leads to endothelial injury, abnormal smooth muscle cell proliferation, and accumulation of oxidized LDL (ox-LDL) in the arterial wall, all of which contribute to the progression of atherosclerosis (179). Endothelial injury represents an early physiological and pathological change in atherosclerosis, accompanied by reduced synthesis and secretion of nitric oxide (NO). NO inhibits platelet aggregation, monocyte adhesion, and smooth muscle cell proliferation. Oxidative stress induced by ROS can inactivate endothelial nitric oxide synthase, leading to decreased NO secretion (180). Dysfunctional endothelial nitric oxide synthase can further promote ROS production, which exacerbates endothelial damage by reducing NO synthesis and secretion. Recent studies have indicated that mitochondrial calcium overload, decreased membrane potential, ROS production, and cytochrome c release are involved in oxLDL-induced endothelial cell apoptosis (181). Overexpression of PGC1α can reduce mitochondrial ROS levels and reverse oxLDL-induced mitochondrial metabolic disturbances. Furthermore, overexpression of PGC1α can reduce inflammation by decreasing the activity of NF-κB and TNF-α (182). Additionally, overexpression of OPA1 promotes mitochondrial autophagy and fusion, restores endothelial cell vitality, and reduces oxLDL-induced apoptosis (183). Vascular smooth muscle cells are crucial components of fatty streaks and plaques. Mitochondrial dysfunction is a notable feature of atherosclerotic vascular smooth muscle cells. Mfn2 plays a significant role in the proliferation and apoptosis of vascular smooth muscle cells. In ApoE-/- mice, Mfn2 expression in vascular smooth muscle cells is significantly reduced, but overexpression of Mfn2 can inhibit abnormal smooth muscle cell proliferation (184). This is because Mfn2 overexpression can mediate smooth muscle cell apoptosis by inhibiting protein kinase B (Akt) signaling and activating mitochondrial apoptosis (185). Further research has shown that Mfn2 overexpression can inhibit oxLDL-induced smooth muscle cell proliferation by suppressing extracellular regulated protein kinases (ERK) and Akt phosphorylation (186). Additionally, mitochondrial DNA damage has been observed early in atherosclerosis, with an increase in heterogeneous mitochondrial DNA variants correlating with the degree of atherosclerotic morphological phenotype (187). ROS-induced mitochondrial DNA damage impairs mitochondrial function, promotes apoptosis of vascular smooth muscle cells and macrophages, inhibits smooth muscle cell proliferation, increases necrotic core size, reduces fibrous cap area, and increases plaque vulnerability. However, restoring mitochondrial DNA integrity can improve these conditions. In atherosclerosis patients, repair of nuclear and mitochondrial DNA in vascular smooth muscle cells requires ATP and reactive oxygen species, leading to further mitochondrial dysfunction, accelerated plaque formation, and impaired smooth muscle cell function.

#### Vascular calcification

Aging is currently associated with chronic low-grade inflammation, which exacerbates age-related diseases. Vascular calcification is a common complication of aging, particularly closely related to atherosclerosis and type 2 diabetes. It has been reported that aging elevates inflammatory cytokine levels, such as interleukin-6, and impairs mitochondrial function in the aorta (188). Studies have shown that vascular calcification is linked to inflammatory states, and mitochondrial dysfunction induced by inflammation can affect the development of vascular calcification. Therefore, the relationship between mitochondrial dysfunction and vascular calcification has been extensively studied. Zhu et al. found that lactate treatment accelerates the calcification process and leads to mitochondrial dysfunction in vascular smooth muscle cells, while BNIP3-mediated mitophagy can restore mitochondrial function and inhibit lactate-induced calcification (189). Rogers et al. (46) also found that Drp1 is highly expressed in calcified human carotid arteries, and inhibition of Drp1 expression can alleviate smooth muscle cell and human valve interstitial cell calcification. Additionally, mitochondria directly participate in the calcification process through reactive oxygen species (ROS) production (190). Furthermore, resveratrol can improve mitochondrial function and oxidative stress via the PKA/LKB1/AMPK pathway. Vascular calcification is a complex, multi-factorial, and prolonged process. Various mechanisms may influence the occurrence and development of vascular calcification through mitochondrial pathways. Despite significant advances in mechanical treatments such as rotational atherectomy, these methods are passive treatments for advanced calcification (191). The association between mitochondrial dysfunction and vascular calcification offers a new approach to prevention and treatment, namely mitochondrial-targeted therapy and antioxidant treatment.

## Mitochondrial targeting in cancer therapy

Targeting mitochondria for cancer therapy represents an emerging treatment strategy that aims to inhibit tumor cell growth and dissemination by directly affecting mitochondrial function. Mitochondria play a crucial role in tumor cells, with their dysfunction often leading to metabolic reprogramming that supports rapid tumor growth and resistance to therapy. Mitochondrial-targeted therapeutic approaches are designed to disrupt mitochondrial function in tumor cells, thereby inducing apoptosis or other forms of cell death (192). These therapeutic strategies include the use of mitochondrial-targeted drugs, mitochondrial-penetrating peptides, and compounds that regulate mitochondrial dynamics and function. For instance, certain drugs can specifically accumulate within mitochondria, interfering with oxidative phosphorylation or increasing the production of reactive oxygen species (ROS), leading to tumor cell death. Additionally, promoting mitochondrial autophagy can clear damaged mitochondria, thereby reducing the survival capacity of tumor cells. Targeting mitochondria not only effectively kills tumor cells but also enhances anti-tumor immune responses. Disruption of mitochondrial function can alter the tumor microenvironment, boosting the activity and tumor-suppressive capabilities of immune cells. Although research in this area is still in its early stages, existing studies demonstrate the substantial potential of mitochondrial-targeted therapies for cancer, providing new directions for the development of novel anti-cancer drugs and treatment strategies (193). Representative drugs targeting mitochondria for cancer therapy include etoposide, betulinic acid, and doxorubicin. These agents exert their anticancer effects by disrupting mitochondrial functions, such as inhibiting the mitochondrial respiratory chain, inducing mitochondria-mediated apoptosis, or disrupting the mitochondrial membrane potential. Furthermore, advancements in mitochondrial-targeted delivery systems (e.g., MITO-Porter) are rapidly evolving, offering new avenues to enhance therapeutic efficacy while reducing toxicity. Such therapies hold significant promise for the treatment of various cancers. Herein, we systematically summarize the currently reported representative drugs targeting mitochondria in cancer therapy and their underlying mechanisms of action (**Table 2**).

**Table 2:** Drugs targeting mitochondria for cancer therapy and their mechanisms of action.

Targets	Drug	Mechanism
Mitochondrial metabolism	metformin	Inhibit mitochondrial complex I, reduce ATP production, activate the AMPK pathway, and thereby inhibit tumor cell proliferation
	phenformin	Inhibit mitochondrial complex I, induce oxidative stress, and promote cancer cell apoptosis
	3-Bromopyruvate	Inhibit mitochondrial oxidative phosphorylation and glycolysis, deplete the energy supply of tumor cells
Mitochondrial membrane	mitoQ	Protect the mitochondrial membrane through antioxidant action and regulate cancer cell survival and apoptosis via ROS
	dichloroacetate	Activate mitochondrial function, reverse the Warburg effect, and enhance cancer cell sensitivity to apoptosis
	venetoclax	Target the BCL-2 protein, increase mitochondrial outer membrane permeability, and induce cell apoptosis
Mitochondrial ROS	arsenic Trioxide	Increase mitochondrial ROS levels, induce oxidative stress, and trigger apoptotic signaling pathways
	elesclomol	Induce cancer cell apoptosis by increasing mitochondrial ROS generation
Mitochondrial DNA	BAY87-2243	Inhibit mitochondrial complex I, interfere with mtDNA replication and expression, and reduce tumor cell proliferation
	Doxorubicin	Bind to mtDNA, disrupt mitochondrial function, and induce cell apoptosis
Mitochondrial apoptotic pathway	obatoclax	Bind to mtDNA, disrupt mitochondrial function, and induce cell apoptosis
	navitoclax	Increase mitochondrial outer membrane permeability by inhibiting BCL-2 family proteins and trigger cell apoptosis

## Inducing mitochondrial ROS dysregulation in tumors

Evidence suggests that hypoxia and oxidative stress can control metabolic reprogramming in cancer cells and other cells within the tumor microenvironment, and reprogrammed metabolic pathways in cancer tissues can alter redox balance. Based on ROS therapy, an effective approach may exploit the characteristics of tumor cells to produce more ROS and antioxidants, inducing ROS dysregulation in tumor mitochondria (194). This approach aims to inhibit antioxidants in tumor cells, ultimately leading to selective tumor cell killing. Targeting mitochondrial antioxidants with drugs could be significant in cancer prevention and treatment. Triphenylphosphonium-doxorubicin (TPP-DOX) can induce ROS dysregulation in tumor cells, characterized by caspase activation, leading to apoptosis. Selective amplification of ROS generation in tumor cells is recognized as an effective strategy for cancer therapy. However, abnormal tumor metabolism, especially mitochondrial glutamine catabolism, can promote high levels of antioxidants in tumor cells to evade ROS-induced damage (36). Research has shown that a tumor-targeting nanoparticle platform combining inhibition of mitochondrial glutamine catabolism and chemotherapeutic agents can treat cancer by blocking nutrient supply and disrupting intracellular redox homeostasis, ultimately inhibiting tumor growth. In summary, targeted drugs inducing mitochondrial ROS dysregulation in tumor cells can have a suppressive effect on tumors.

### Targeting mtDNA for cancer therapy

Mitochondrial DNA (mtDNA) is the only remaining extranuclear genetic material in cells, and the genes encoded within mtDNA can lead to mitochondrial diseases and malignancies. Notably, the D-LOOP region in the mammalian mitochondrial genome is crucial for mtDNA replication (6). Anticancer drugs that target mitochondria can interact with relevant DNA, further validating their role in genetic material replication and transcription, and guiding the selection of gene-related anticancer drugs (195). Studies have shown that mtDNA mutations are associated with small-cell lung cancer and multidrug resistance, and variations in mitochondrial DNA copy number are closely related to gastric cancer risk assessment. Some scholars believe that mtDNA copy number and telomere length are significantly associated with cancer incidence and mortality, though these associations vary by cancer type and require further investigation (196). Research on primary and metastatic uveal melanoma has shown that mtDNA copy number is significantly increased in metastatic uveal melanoma compared to primary uveal melanoma, which is closely linked to its migration and invasion (197). Studies indicate that polyphenols in pomegranate fruit can induce mtDNA damage and even nuclear DNA damage in oral cancer cells, promoting apoptosis and inhibiting tumor cell proliferation (198). Yeung et al. (12) found that bleomycin can induce mtDNA damage, making it a potentially effective drug for targeting mtDNA in acute myeloid leukemia (199). Exploring the relationship between mtDNA and tumors and targeting drugs will pave the way for advances in medical treatment.

### Targeting the bcl-2 protein family to induce apoptosis

The Bcl-2 protein family is crucial in regulating apoptosis, comprising pro-apoptotic and anti-apoptotic proteins. Bcl-2 is one of the most potent anti-apoptotic factors, whereas Bax is a major pro-apoptotic protein, and their balance maintains cellular stability (200). Natural compound oroxylin A can reduce Bcl-2 expression and induce mitochondrial-mediated apoptosis, thus inhibiting colon cancer cell proliferation and migration (201). Literature indicates that α-tocopheryl succinate (α-TOS), a redox-silent analog of vitamin E, can inhibit the interaction of Bak’s BH3 domain with Bcl-2 and Bcl-xL, weakening their biological activity and increasing mitochondrial outer membrane permeability, leading to apoptosis in breast and colon cancer cells (202). In non-small cell lung cancer, a non-chromosomal structural maintenance protein complex subunit I G (NCAPG) regulates cell division. Downregulation of NCAPG promotes the expression of caspase-3 and Bax while inhibiting anti-apoptotic protein Bcl-2 expression, thereby promoting mitochondrial-related apoptosis in tumor cells (203). Interestingly, recent research shows that mango peel extract can inhibit cell viability in colon cancer cell lines, affecting the Bcl-2 protein family and apoptosis factors while causing mitochondrial damage, offering a potential new strategy for cancer treatment (204). Besides the increased mitochondrial outer membrane permeability caused by Bcl-2 protein family interactions and the BH3 domain, increased permeability due to the mitochondrial permeability transition pore can also effectively play an anti-tumor role (205). These studies suggest that drugs targeting the Bcl-2 protein family can activate the apoptotic system, promote cell apoptosis, and ultimately exert anti-tumor effects, potentially providing more effective and novel treatment options for cancer.

### Targeting the inhibition of ATP production in tumor cell mitochondria

Mitochondria are the primary sites for cellular energy metabolism and aerobic respiration, utilizing oxidative phosphorylation or the electron transport chain to generate adenosine triphosphate (ATP) from carbohydrates. ATP is considered a potential strategy for tumor prevention or a carrier for tumor-targeting drugs (206). However, due to the rapid degradation and brief presence of ATP outside cells, current detection methods have limitations (207). Studies have shown that pomegranate polyphenol extracts can induce ATP depletion and disrupt mitochondrial membrane potential in three types of oral cancer cells, inhibiting proliferation and inducing apoptosis via mitochondrial pathways (208). Metformin can also target ATP production, impairing respiratory chain function and inhibiting the growth of insulin-dependent tumor cells, though the mechanisms of metformin’s action and its clinical dosing are still under investigation (209). Research indicates that metformin can enhance autophagy, thereby protecting neuronal cells from aging and apoptotic neurodegenerative changes (210). Some scholars have identified lichen compounds that inhibit the proliferation and metastasis of breast cancer cells, including triple-negative breast cancer. These compounds affect mitochondrial energy metabolism, impairing aerobic respiration and glycolysis, reducing ATP production, and further inhibiting tumor growth (211). Monitoring ATP levels in live cells is crucial for the clinical diagnosis of various diseases, including cancer. Therefore, future experimental research on tumors could focus on targeting mitochondrial ATP production inhibition.

### Targeting mitochondrial autophagy to inhibit tumor biosynthetic pathways

Autophagy is a critical process for maintaining cellular homeostasis and participating in metabolic breakdown, playing important roles in anti-aging, immunity, anti-tumor responses, and stress resistance under complex conditions (212). In research on liver malignancies, it has been found that mitochondrial autophagy is crucial for cell growth, making autophagy a new target for cancer treatment (213). Mitochondria sustain normal cellular activities through fatty acid metabolism, the tricarboxylic acid cycle, and oxidative phosphorylation, and can also be influenced by various factors to initiate apoptosis (214). During tumor cell biosynthesis, hexokinase II, involved in glycolysis, is highly expressed and can bind to voltage-dependent anion channels. Research on anti-tumor drugs could focus on preventing the interaction between hexokinase II and voltage-dependent anion channels (215). Studies have shown that the active compound A-24 extracted from onions can regulate autophagy and apoptosis via the PIK/Akt/mTOR signaling pathway, significantly inhibiting proliferation in wild-type p53 gastric cancer cells (216). Among heat-clearing and detoxifying herbs, half-leaf and white flower snake tongue herbs exhibit anti-inflammatory, anti-tumor, and free radical scavenging effects, and are used in anti-tumor formulations (217)]. In one study, the ethyl acetate fraction of extracts from white flower snake tongue herb and half-leaf significantly increased the inhibition rate of gastric cancer cell proliferation, demonstrating its anti-cancer effects, which are closely related to mitochondrial autophagy (218). Additionally, high-phenol sorghum bran extract can inhibit cellular biosynthetic pathways by activating autophagy and related signaling pathways, ultimately suppressing colorectal cancer formation (219). Apoptosis and autophagy are critical for cellular life activities. Targeting mitochondrial biosynthetic pathways can activate apoptosis signaling pathways, thereby inhibiting cancer cell proliferation and metastasis, and inducing cancer cell apoptosis.

### Limitations of mitochondrial-targeted therapies

Mitochondrial-targeted therapies, such as ROS modulators and mitophagy enhancers, have demonstrated significant potential in the treatment of cancer and other diseases. These therapies aim to restore intracellular homeostasis by modulating mitochondrial function, thereby inhibiting tumor growth or mitigating disease progression. For instance, ROS modulators help balance redox states to prevent oxidative stress-induced cellular damage, while mitophagy enhancers clear damaged mitochondria, reducing their toxic accumulation within cells. However, several challenges remain in the clinical application of these therapies. These include insufficient drug specificity, difficulties in achieving efficient mitochondrial delivery, the delicate balance between therapeutic dosage and toxicity, and variability in patient responses due to individual differences. Additionally, mitochondrial-targeted therapies may inadvertently affect the functions of healthy tissues, raising concerns about their safety profiles. Thus, despite their promising prospects, the clinical translation of mitochondrial-targeted therapies requires further investigation to overcome these challenges and optimize their therapeutic efficacy.

## Conclusion

Mitochondria play a central role in cellular function, participating in energy metabolism, signal transduction, and metabolic regulation. Mitochondria generate ATP through oxidative phosphorylation, providing the primary energy source for cells, while also producing reactive oxygen species (ROS) and regulating the redox state within cells. Mitochondrial quality control is primarily achieved through mitophagy and the dynamic balance of fusion and fission, ensuring that damaged mitochondria are effectively removed and maintaining mitochondrial functional integrity (220). Mitochondrial biogenesis is regulated by nuclear-encoded proteins and the mitochondria’s own DNA (mtDNA). mtDNA is a circular double-stranded DNA encoding essential components of the respiratory chain complexes, tRNA, and rRNA. Mutations or damage to mtDNA can directly impact mitochondrial function, leading to various metabolic diseases (221). The stability of the mitochondrial genome is crucial for cellular health, and mechanisms for mtDNA replication and repair play a key role in this process. Mitochondrial function is closely related to health and longevity. Maintaining mitochondrial function is vital for delaying the cellular aging process. With aging, mitochondrial function declines and the accumulation of ROS accelerates cellular aging. Enhancing mitochondrial function or removing damaged mitochondria can extend cellular lifespan. In terms of regulating cell death, mitochondria initiate apoptosis by releasing cytochrome c and other pro-apoptotic factors and play significant roles in ferroptosis and pyroptosis (222). Mitochondria often referred to as the powerhouses of the cell, play a crucial role in maintaining cellular function by providing energy and regulating various physiological and pathological processes. These organelles are deeply involved in cellular dynamics, metabolic regulation, and cell fate determination. Dysfunctional mitochondria are linked to numerous diseases, including autoimmune disorders, neurodegenerative diseases, and cancer. Despite significant advances in understanding mitochondrial function, the complexity of these functions and the disturbances caused by their defects remain critical issues. It is essential to unveil new mitochondrial functions at the molecular level and develop animal models for mitochondrial diseases.

Mitochondria generate large amounts of ATP, which supplies cells with the energy required for life activities. They also play vital roles in the biosynthesis of macromolecules, cellular signal transduction, immune cell activation, and cell fate determination. This paper explores mitochondrial metabolism and biosynthesis, focusing on the intricate interactions between key mitochondrial signaling components (such as mtDNA and mtROS), the tumor microenvironment, and the immune system. The aim is to provide insights into the development of novel anti-tumor immunotherapies from the perspective of mitochondria and tumor immunity. Mitochondrial energy metabolism and biosynthesis are crucial in immune cell activation. However, tumor cells’ competitive glucose consumption and the hypoxic tumor microenvironment induce mitochondrial damage and excessive ROS production, resulting in immune cells enduring metabolic insufficiency and high oxidative stress. This environment disrupts immune cell activation and tumor immunosurveillance, leading to tumor immune escape. Therefore, combining metabolic therapy with immune checkpoint inhibitors by enhancing immune cell energy metabolism, reducing mitochondrial dysfunction and mtROS production in immune cells, and increasing the survival of effector T cells and the generation of memory T cells is a novel strategy to control tumor growth.

Moreover, *ex vivo* mtDNA editing of chimeric antigen receptor (CAR) T cells can promote mitochondrial biosynthesis and oxidative phosphorylation, compensating for the progressive loss of tumor-infiltrating effector T cells during anti-tumor immune responses and enhancing the anti-tumor immunity of immune cells. Additionally, radiotherapy can induce mitochondrial dysfunction and ROS outbursts, leading to tumor cell apoptotic cascades, which is a significant direction for anti-tumor immunotherapy research and clinical practice. MtDNA damage, mutations, and cytoplasmic leakage are critical factors in the development of various diseases, including cancer. During cellular damage, cytoplasmic mtDNA serves as an important signaling molecule that activates innate immune signaling pathways. Under physiological conditions, mtDNA exists in a nucleoid structure within the mitochondrial matrix. Upon cellular damage, mitochondria undergo outer membrane permeabilization or mitochondrial permeability transition pore opening, mediating mtDNA entry into the cytoplasm and activating multiple innate immune signals, including NLRP3, TLR9, and cGAS-STING, triggering inflammatory responses and cell death. Notably, while apoptosis is typically immune-silent, under certain conditions, such as caspase activity inhibition by drugs, mtDNA-mediated inflammatory responses are significantly enhanced, promoting immunogenic cell death and boosting the host’s anti-tumor immune response.

Targeting mtDNA has become a promising cancer treatment strategy. The response mechanisms following mtDNA damage are crucial for developing mtDNA-targeted drugs, predicting therapeutic efficacy, and analyzing potential resistance. Although mitochondria play a central regulatory role in the complex interplay between cancer and the immune system, therapeutic strategies that modulate the host’s anti-tumor immunity by targeting mitochondria are currently a focal point of basic and clinical research. However, these studies are still in their early stages. Investigating how mitochondrial activity and biological functions are regulated during the activation of different cell subpopulations, shifting energy balance toward immune cells, and promoting the repair of mitochondrial dysfunction and metabolic insufficiency in immune cells while inhibiting tumor cell metabolism are crucial research directions. These will become vital components in improving anti-tumor efficacy in clinical patients.

In the context of tumor progression, cancer cells often undergo metabolic reprogramming to meet the demands of rapid growth and proliferation, with mitochondrial dysfunction potentially promoting malignant transformation and metastasis. The role of mitochondria in the tumor immune microenvironment is also of significant interest, as mitochondrial metabolic abnormalities in the tumor microenvironment can influence anti-tumor immune responses by regulating the function and activation of immune cells. Metabolites and signaling molecules produced by mitochondria play critical roles in the polarization and functional regulation of immune cells. Mitochondrial metabolic abnormalities are closely related to various diseases, including neurodegenerative diseases, cardiovascular diseases, metabolic disorders, and cancer. Mitochondrial dysfunction leads to insufficient ATP production, excessive ROS generation, and metabolic imbalance within cells, ultimately causing cellular damage and disease (223). Understanding the role of mitochondria in these diseases is crucial for developing therapeutic strategies targeting mitochondrial dysfunction.

## Future research directions

### Mitochondrial immune regulation

Mitochondria play a crucial role in immune regulation, serving as both platforms for immune signaling and centers for metabolic control, which has garnered significant attention. For instance, ROS and metabolic byproducts produced by mitochondria act as key signaling molecules that regulate the activation and function of immune cells. Additionally, the release of mitochondrial DNA into the cytoplasm can activate the cGAS-STING pathway, triggering innate immune responses. In the future, investigating how mitochondria interact with immune checkpoints in the immune microenvironment may offer new strategies for cancer immunotherapy and advance the treatment of autoimmune diseases.

### Mitochondrial spatial transcriptomics

With the rapid development of single-cell spatial transcriptomics technology, it has become possible to study the dynamic distribution of the mitochondrial transcriptome within tissue microenvironments. By analyzing the spatial heterogeneity of mitochondrial gene expression, specific functions of mitochondria in different cell types or subpopulations can be revealed. For example, the spatial characteristics of mitochondrial gene expression in tumor tissues may be closely associated with tumor progression and therapeutic resistance. Future research in this field will contribute to the construction of mitochondrial transcriptional networks in diseases, offering new targets for precision medicine.

### Mitochondrial epitranscriptomics

Mitochondrial epitranscriptomics is an emerging research field that primarily explores the regulatory effects of mitochondrial RNA modifications (such as m6A, m5C) on its function and stability. These modifications not only affect the translation efficiency and degradation of mitochondrial RNA but may also regulate the communication between mitochondria and the nucleus. In the future, understanding the molecular mechanisms of mitochondrial RNA modifications and their roles in diseases will aid in developing therapeutic strategies based on epitranscriptomics.

### Mitochondrial genome editing and replacement therapies

Mitochondrial genome editing technologies (such as modified TALEN and CRISPR-Cas9) are opening up new possibilities for the treatment of mitochondrial-related genetic diseases. These technologies can precisely edit mitochondrial DNA to correct pathogenic mutations and restore mitochondrial function. Additionally, mitochondrial replacement therapies (such as mitochondrial replacement therapy used in three-parent embryos) show great potential in preventing maternally inherited diseases. Although these technologies are still in the early stages, they hold promise for revolutionizing the treatment of mitochondrial diseases and certain complex disorders once safety and ethical concerns are addressed. In conclusion, the future direction of mitochondrial research will be increasingly diverse and precise. Through interdisciplinary exploration, it is expected to pave new paths for the diagnosis, treatment, and prevention of diseases.
